# A new approach for the assessment of the toxicity of polyphenol-rich compounds with the use of high content screening analysis

**DOI:** 10.1371/journal.pone.0180022

**Published:** 2017-06-29

**Authors:** Magdalena Boncler, Jacek Golanski, Magdalena Lukasiak, Malgorzata Redzynia, Jaroslaw Dastych, Cezary Watala

**Affiliations:** 1 Department of Haemostasis and Haemostatic Disorders, Medical University of Lodz, Lodz, Poland; 2 Proteon Pharmaceuticals SA, Lodz, Poland; 3 Institute of Technical Biochemistry, Faculty of Biotechnology and Food Sciences, Lodz University of Technology, Lodz, Poland; 4 Laboratory of Cellular Immunology, Institute of Medical Biology, Polish Academy of Sciences, Lodz, Poland; University of South Alabama Mitchell Cancer Institute, UNITED STATES

## Abstract

The toxicity of *in vitro* tested compounds is usually evaluated based on AC_50_ values calculated from dose-response curves. However, there is a large group of compounds for which a standard four-parametric sigmoid curve fitting may be inappropriate for estimating AC_50_. In the present study, 22 polyphenol-rich compounds were prioritized from the least to the most toxic based on the total area under and over the dose-response curves (AUOC) in relation to baselines. The studied compounds were ranked across three key cell indicators (mitochondrial membrane potential, cell membrane integrity and nuclear size) in a panel of five cell lines (HepG2, Caco-2, A549, HMEC-1, and 3T3), using a high-content screening (HCS) assay. Regarding AUOC score values, naringin (negative control) was the least toxic phenolic compound. Aronox, spent hop extract and kale leaf extract had very low cytotoxicity with regard to mitochondrial membrane potential and cell membrane integrity, as well as nuclear morphology (nuclear area). Kaempferol (positive control) exerted strong cytotoxic effects on the mitochondrial and nuclear compartments. Extracts from buckthorn bark, walnut husk and hollyhock flower were highly cytotoxic with regard to the mitochondrion and cell membrane, but not the nucleus. We propose an alternative algorithm for the screening of a large number of agents and for identifying those with adverse cellular effects at an early stage of drug discovery, using high content screening analysis. This approach should be recommended for series of compounds producing a non-sigmoidal cell response, and for agents with unknown toxicity or mechanisms of action.

## Introduction

Plant polyphenols constitute a highly heterogeneous group of compounds which play a plethora of physiological and ecological roles in plants. Some phenolic compounds produced by plant tissues, like flavonoids, are widely distributed in the plant kingdom, but others are often restricted to specific genera or even families, making them convenient biomarkers for taxonomic studies [1]. Flavonoids demonstrate important effects in plant biochemistry and physiology, acting as antioxidants, enzyme inhibitors, and precursors of toxic substances. In addition, they are involved in photosensitization and energy transfer, respiration, photosynthesis, regulation of plant growth, and defense against infections [2]. Numerous herbal remedies containing flavonoids have been used in traditional Eastern medicine for thousands of years. They have long been recognized to possess anti-inflammatory, antioxidant, anti-allergic, hepatoprotective, antiviral, cardioprotective and anti-cancer activities [2]. This wide range of activities clearly demonstrates the huge pharmacological potential of plants for the pharmaceutical industry. Due to the development of treatment-related complications, such as drug resistance and adverse effects, natural compounds have been often suggested to offer new, alternative therapeutic strategies, either to complement or to replace existing conventional medicine approaches.

Toxicity testing of new compounds is essential for the drug development process. There are numerous conventional cytotoxicity methods which allow the *in vitro* effects of new drug candidates to be examined on living cells. The basic cytotoxic tests include those that measure metabolic activity of the cells, plasma membrane integrity, changes in cell number and morphology, cell growth/proliferation or the mechanisms of cell death [3]. However, one major limitation of this kind of assay is their inability to measure a wide spectrum of potential early or late pathological changes involved in drug-induced toxic injury. Most conventional tests evaluate only one endpoint, whereas multiple mechanisms of toxicity would need to be verified by multiple assays involving the use of morphological, biochemical or functional parameters. Furthermore, the measurements would need to be performed directly at the individual cell level in order to minimize artefacts and to ensure that they truly reflect cell-associated effects [4].

An important breakthrough in this field was achieved in 1997, when high content screening (HCS) technology, also known as high content analysis (HCA), was introduced into the market as a new, effective tool for the assessment of toxicity [5]. High content screening is defined as a combination of modern cell biology, automated high-resolution microscopy and flow cytometry integrated to simultaneously detect multiple parameters, such as nuclear area/intensity, intracellular calcium level, mitochondrial membrane potential, plasma membrane permeability and cell number. The process allows the characterization of new therapeutic lead compounds and the identification of the mechanisms of drug toxicity, including mitochondrial dysfunction, oxidative stress, calcium dyshomeostasis, apoptosis, phospholipidosis and steatosis [6,7]. In addition, HCS may serve as an important predictive tool for the optimization and prioritization of the safety of a compound [4,8].

As metabolically competent cells, human primary hepatocytes and the hepatoma HepG2 cell line provide the closest *in vitro* models to human liver and hence they are the most appropriate candidates for use in cytotoxicity assays. Studies using human hepatocytes have shown that HCS is more effective than past methods for identifying cytotoxic substances in humans [4]. The ability of HCS to quickly analyze the toxicological effects of a large number of compounds has also been confirmed in other studies, using various cell types, such as macrophages [9,10], human bronchial epithelial cells [10], human monocytes [11], intestinal epithelial cells [11], neuronal cell lines [12], lymphocytes [7], human osteosarcoma cells [13], and bovine kidney cells [14]. As a result of global acceptance of HCS technology in drug discovery and basic biomedical research [6], recent years have seen a growing number of reports dealing with the evaluation of the toxicity of various compounds, including drugs with known clinical hepatotoxicity profiles [15], fungal toxins occurring in foods [14], nanoparticles [16] and plant extracts [17,18].

In the present study, HCS was used to prioritize nineteen plant extracts together with three well-known and widely-described phytochemicals, based on their toxicity towards five cell lines. As natural compounds can trigger various responses, it is not always possible to determine toxicity by estimating the AC_50_ value and the maximum effect. Hence, the present study presents an alternative algorithm to estimate the toxicity of polyphenol-rich compounds and prioritize them. This new approach allows the assessment of the degree of cytotoxicity of an unlimited number of compounds with potential health impacts. High content screening provides a powerful and reliable tool for the fast analysis of plant compound toxicity against different target cell lines.

## Materials and methods

### Chemicals and cell lines

The HepG2, Caco-2, A549, and 3T3 cell lines (the European Collection of Cell Cultures; ECACC) were obtained from Sigma Chemical Co. (St. Louis, MO, USA), while the HMEC-1 cell line was obtained from ATCC (Salisbury, UK). L-glutamine was obtained from PAA (Pasching, Austria). Cell culture media, such as DMEM and MCDB 131 with supplements, were obtained respectively from GIBCO (Carlsbad, CA, USA) and Cytogen (Sinn, Germany). MitoTracker and YO-PRO 1 were from Invitrogen (Carlsbad, CA, USA). Collagen (Cultrex Rat Collagen I) was obtained from R&D Systems (Minneapolis, MN, USA). All solvents of reagent or analytical grade were purchased from POCH (Gliwice, Poland). All the remaining reagents were purchased from Sigma Chemical Co. (St. Louis, MO, USA). Plastic disposable flasks, 96-well tissue culture plates, pipettes, and tubes were purchased from *Nunc* (Roskilde, Denmark).

### Plant material

The study used seventeen freshly prepared plant extracts, two commercial plant extracts (Aronox and Omnivir R), and three natural phenolic compounds of plant origin which are regularly consumed by humans: naringin, kaempferol and resveratrol.

Willow (*Salix* L.) bark, oak (*Quercus* L.) bark, buckthorn (*Frangula alnus* Mill.) bark, hollyhock (*Alcea rosea* L. *var*. *nigra*) flowers, mallow (*Malva* L.) flowers and silverweed (*Potentilla anserina* L.) herb were supplied by the Kawon-Hurt Nowak Sp. J. (Gostyń, Poland). Spent hop (*Humulus lupulus* L.) extract, after the hop extraction with supercritical CO_2_, was supplied by the Fertilizer Research Institute Puławy (Poland). Black currant (*Ribes nigrum* L.) pomace was supplied by Agros Nova Sp. z o. o. (Łowicz, Poland). Raspberry (*Rubus idaeus* L.) seeds were separated from the fruits of the *Polka* variety. Red kale (*Brassica oleracea* L. *var*. *sabellica*) was purchased in a retail trade in Lodz. Other raw plant materials, such as walnut (*Juglans regia* L.) husks, rowan (*Sorbus aucuparia* L.) fruits, birch (*Betula verrucosa* Ehrh.) inflorescences and the leaves of black currant (*Ribes nigrum* L.), blackberry (*Rubus fructicosus* L.), oak (*Quercus robur* L.), and birch (*Betula verrucosa* Ehrh.) were collected from the natural environment near Lodz.

Aronox (Adamed, Poland) is a supplement produced from chokeberry fruits (*Aronia melanocarpa* (Michx.) Elliott), while Omnivir R (C.E. Roeper GmbH, Germany) is produced from grape seeds (*Vitis vinifera* L.). Naringin, kaempferol and resveratrol have been experimentally documented to possess numerous biological properties and potential therapeutic applications [19–21]. All are recommended as reference standards by the European Pharmacopoeia (naringin, resveratrol) or United States Pharmacopeia (kaempferol) (https://www.sigmaaldrich.com). Resveratrol was used along with the plant extracts, whereas naringin and kaempferol served as negative and positive controls, respectively. Naringin, which exerts a lower cytotoxic effect than kaempferol against various cells [22–27], was used at half the micromolar concentration as kaempferol (*see below*).

### Preparation of polyphenolic extracts and determination of total phenolic content

Solid polyphenolic extracts were obtained as previously described [28]. Liquid polyphenolic extracts were prepared from dried or frozen homogenized materials. These were extracted in acetone-water (70:30, v/v) at a solid to liquid ratio of 1:10 (w/v) for 30 minutes at RT and then centrifuged at 4,000 rpm for 15 minutes. The residues were re-extracted twice with 70% aqueous acetone at a solid to liquid ratio 1:5 (w/v) for 15 minutes and the supernatants were combined. The extracts were then evaporated at <40°C under reduced pressure (Rotavapor RII, Büchi, Switzerland) and the water solutions were extracted with chloroform (2- to 8-fold, 1:1 v/v). Finally, the defatted aqueous extracts were evaporated and made up to 30 ml with water containing DMSO (final concentration 10% v/v). After centrifugation (4,000 rpm, 10 minutes), the obtained extracts were frozen at −24°C until analysis. Total phenolic content was determined using the Folin-Ciocalteu method [29]. Phenolic content was estimated from a standard curve of gallic acid. More details on the extraction process and the total phenolic content are provided in Table 1.

**Table 1:** Characteristics of extraction procedure and total phenolic content in the examined plant extracts.

Common name	Botanical name	Mass of raw material [g]	Number of extractions with chloroform	Extract mass [g] ^a^volume [ml]	Total polyphenol content [mg/g or mg/ml^b^]
**Birch inflorescences**	*Betula verrucosa* Ehrh.	20	4	1.7	454.5 ± 20.0
**Birch leaves**	*Betula verrucosa* Ehrh.	20	7	30^a^	19.4 ± 1.0^b^
**Black currant leaves**	*Ribes nigrum* L.	10	3	2.2	267.7 ± 19.2
**Black currant pomace**	*Ribes nigrum* L.	100	2	2.2	86.3 ± 2.2
**Blackberry leaves**	*Rubus fructicosus* L.	10	3	1.7	301.8 ± 24.2
**Buckthorn bark**	*Frangula alnus* Mill.	20	8	2.9	142.6 ± 6.4
**Hollyhock flowers**	*Alcea rosea* L. *var*. *nigra*	20	3	30^a^	12.4 ± 1.0^b^
**Kale leaves**	*Brassica oleracea* L. *var*. *sabellica*	50	4	2.6	75.0 ± 5.9
**Mallow flowers**	*Malva* L.	20	5	30^a^	13.2 ± 1.3^b^
**Oak bark**	*Quercus* L.	20	3	2.2	494.0 ± 17.7
**Oak leaves**	*Quercus robur* L.	10	6	1.4	147.9 ± 7.2
**Raspberry seeds**	*Rubus idaeus* L.	20	2	0.9	263.3 ± 16.3
**Rowan fruits**	*Sorbus aucuparia* L.	50	8	30^a^	12.1 ± 1.0^b^
**Silverweed herb**	*Potentilla anserina* L.	10	2	2.7	498.3 ± 25.0
**Spent hops**	*Humulus lupulus* L.	10	4	1.9	232.9 ± 11.9
**Walnut husks**	*Juglans regia* L.	10	4	1.7	166.4 ± 1.9
**Willow bark**	*Salix* L.	10	2	1.6	271.5 ± 13.5

The values of total polyphenol content, presented as means ± SD (n≥3), are expressed in gallic acid equivalents. For further details see ‘Materials and methods‘.

The solutions of plant extracts in DMSO, freshly prepared for each experiment, were diluted in complete culture medium and added to the cells to make final concentrations of 5, 20, 30, 40, and 50 μg/ml of gallic acid. Solutions of naringin, kaempferol and resveratrol were prepared with the same weight concentrations as in the case of the extracts and they corresponded to 9–86 μM of naringin, 18–175 μM of kaempferol, and 22–219 μM of resveratrol. The concentration of DMSO in the assay (experimental and control samples) never exceeded 0.1% and had no influence on cell growth.

### Cell culture and treatment with polyphenols

The following cell lines: HepG2 (human liver carcinoma cells), Caco-2 (human epithelial colorectal adenocarcinoma cells), A549 (human lung adenocarcinoma epithelial cells), and 3T3 (embryonic mouse fibroblasts) were cultured in DMEM with GlutaMAX supplemented with 10% FBS, 100 U/ml penicillin and 100 μg/ml streptomycin. The HMEC-1 cell line (human microvascular endothelial cells) was cultured in MCDB 131 supplemented with 10% FBS, 100 U/ml penicillin, 100 μg/ml streptomycin, 2 mM L-glutamine, 10 ng/ml epidermal growth factor, and 1 μg/ml hydrocortisone. All cell lines were cultured in a humidified atmosphere with 5% CO_2_ at 37°C in 75 cm^2^ flasks. When the cells reached at least 70% confluence, they were rinsed with phosphate-buffered saline (PBS), detached from the flask by brief exposure to trypsin-EDTA solution, and counted. The wells of 96-well microplates were inoculated with 3,500 HepG2 cells, 10,000 Caco-2 cells, 3,500 A549 cells, 5,000 HMEC-1 cells or 5,000 3T3 cells suspended in 100 μl of suitable culture medium. In the case of HepG2 and Caco-2 cells, collagen-coated plates were used. After 24 hours, the cells were incubated for another 24 hours with polyphenol-rich compounds at final concentrations of 5, 20, 30, 40, and 50 μg/ml. Accordingly, the negative control wells contained 9–86 μM naringin, while the positive control wells included 18–175 μM kaempferol. The control wells contained medium alone.

### High content screening assay

The effects of polyphenol compounds on selected cellular functions, such as mitochondrial membrane potential, cell membrane integrity and nuclear area, were measured by image cytometry, in which data was analyzed simultaneously at single-cell resolution. Fluorescent dyes were combined according to their optical compatibility in a flow cytometer. Mitochondrial membrane potential and cell permeability were measured respectively with MitoTracker and YO-PRO 1. The nuclei of treated cells were stained with Hoechst 33342 dye. Where applicable, media, cells and reagents were added by a JANUS Automated Workstation (Perkin Elmer, Waltham, MA, USA); washing steps and the addition of fluorescent dyes were performed by an ELx405 Microplate Washer (Bio-Tek, Vinooski, VT, USA).

**Fig 1:**
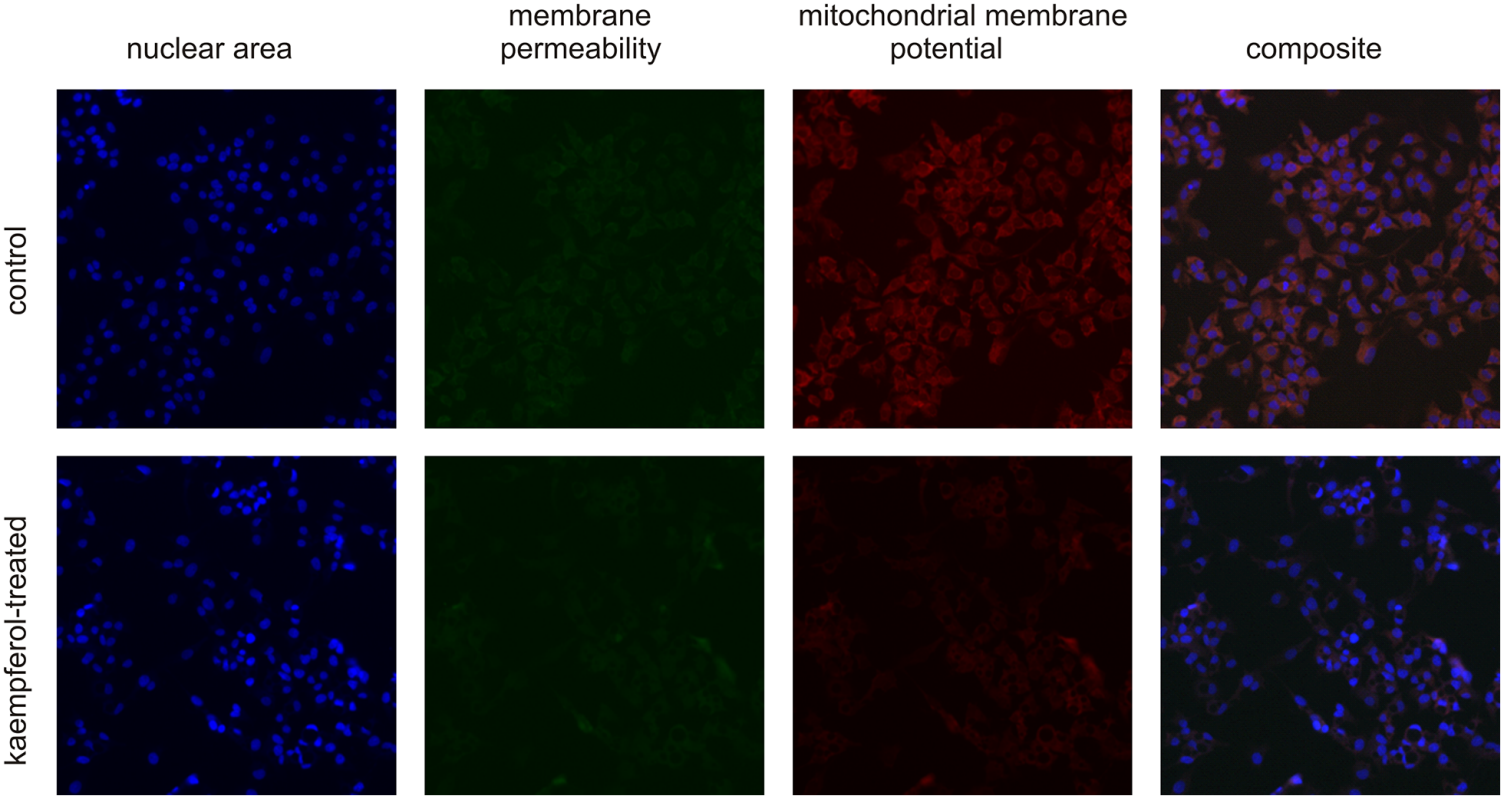
Typical HCS images showing the effects of kaempferol on mitochondrial membrane potential, cell membrane permeability and nuclear area in HepG2 cells. Collagen-coated wells of 96-well microplates were inoculated with 3,500 HepG2 cells. Following 24-hour incubation, the cells were treated with 175 μM kaempferol or vehicle (culture medium with DMSO) and incubated for the next 24 hours. Images of cells stained with YO-PRO 1, MitoTracker, and Hoechst 33342 were acquired with a Cellomics ArrayScan HCS Reader in order to assess the changes in mitochondrial membrane potential, cell membrane permeability and nuclear area.

Briefly, half an hour before the end of the 24-hour incubation with the polyphenol-rich compounds, MitoTracker and YO-PRO 1 were added to the culture medium at final concentrations of 0.1 and 1 μM respectively. Then, the cells were washed twice with PBS and fixed with 2% formaldehyde for 20 minutes at RT. Next, the fixed cells were washed three times with PBS and labeled with Hoechst 33342 (final concentration 1 μM) for 30 minutes in the dark. After washing, the 96-well microplates were measured using Cellomics Array Scan HCS Reader (Thermo Fisher Scientific, Waltham, MA, USA). Measurements were performed using three channels with the following filters: XF93-Hoechst (Channel 1) for Hoechst 33342, XF93-FITC (Channel 2) for YO-PRO 1 and XF93-TRITC (Channel 3) for MitoTracker. Nuclear fluorescence was used for automatic focusing. To measure changes in mitochondrial membrane potential, cell membrane integrity and nuclear area evoked by polyphenolic compounds, the assay protocol was configured to analyze 5000 cells per well. The nuclear area (channel 1) and fluorescence intensities (channel 2 and 3) of single cells were averaged from duplicate wells and the mean nuclear area and fluorescence intensity collected from five independent experiments were quantified. Images were acquired at 10x magnification and analyzed using Multiparameter Cytotoxicity BioApplication V3 software (Cellomics BioApplications; Cellomics, Inc., Pittsburgh, PA, USA). Fig 1 shows typical images derived of the employed immunocytochemistry assays.

### Statistical analysis

Data was collected in duplicate from three cytotoxicity assays: mitochondrial membrane potential, membrane integrity and nuclear area. These tests were performed in five cell lines: HepG2, Caco-2, A549, HMEC-1, and 3T3. All three assays were performed in five independent experiments after the incubation of cells with 5–50 μg/ml plant extracts. In parallel, the cells were incubated with medium alone (baseline), 5–50 μg/ml naringin (9–86 μM; negative control) or 5–50 μg/ml kaempferol (18–175 μM; positive control). To evaluate the toxicity of each polyphenol-rich compound, the areas under and over the curves (AUOC) were calculated in relation to relevant baselines. Such an approach was based on the observation that some concentration-response curves were not sigmoidal and hence, it was impossible to estimate the parameters of the four-parameter non-linear logistic equation. The individual AUOC values obtained for the examined extracts and control phytochemicals were normalized using the van der Waerden score method and averaged for each assay within each cell line. The mean AUOC scores were then placed in ascending order based on the method of measurement (assay) or the cell line, thus producing a ranking of nineteen plant extracts and three phytochemicals (naringin, kaempferol, and resveratrol) in ascending order of toxicity.

The sensitivity of cell lines to polyphenol-rich compounds for each assay was estimated by counting the number of mean AUOC score values above 0, which was directly proportional to cell sensitivity. The total sensitivity of the cell line was calculated by summing the counts for all assays. In the same way, the sensitivity of the assays was determined for the given polyphenol-rich compounds within each of five tested cell lines.

To determine the least and the most toxic polyphenol-rich compound in the assay, regardless of cell line, the cumulative AUOC scores were estimated for each polyphenol-rich compound by summing the mean AUOC scores determined for each polyphenol-rich compound within each of five tested cell lines. In addition, regardless of the assay, the cumulative AUOC scores were estimated for each polyphenol-rich compound with regard to each cell line (HepG2, Caco-2, A549, HMEC-1, and 3T3) by summing up the mean AUOC scores for each of the three assays. The least and most toxic polyphenol-rich compounds in the study were identified based on the global AUOC score. The global AUOC score for a tested compound was estimated by summing up of the cumulative AUOC scores for each of five cell lines, calculated across all three assays. Thus, evaluated measures of cytotoxicity were referred to as cumulative or global averaged normalized AUOC values. The example of the calculation of cumulative and global AUOC scores is given in S1 Table.

In addition, the individual van der Waerden normal scores were also calculated based on individual AUOC values for each set of data, which were further used for inference testing. Furthermore, for each cell line, the cumulative scores (for mitochondrial potential, membrane integrity and nuclear area) were calculated based on five individual score values. The results were tested for normality and variance homogeneity. As the particular data did not meet the assumptions of normality and/or variance homogeneity, the Box-Cox transformation was used. To discriminate between the effects of various examined polyphenol-rich compounds, one-way ANOVA and block ANOVA, followed by the *post hoc* multiple comparison Fisher’s least significant difference test were used to evaluate the statistical differences between the examined extracts and the control reference compounds (naringin and kaempferol). Block ANOVA was employed to demonstrate the impact of two grouping co-variates, an assay and a cell line, on the discrimination between the tested polyphenol-rich compounds, and to compare the extents of such discrimination without and upon the adjustment. The values of Fisher-Snedecore’s statistics (*F*_ANOVA_) were used for these comparisons: a higher *F*_ANOVA_ value indicated better discrimination power, with the polyphenol-rich compound as a grouping variable.

As these approaches for estimating of cytotoxicity indices produced slightly different rankings of toxicity, a few methods were used to compare them: the AC1 Gwet’s test for two raters, the one-tailed Cuzick’s test (which provides a Wilcoxon-type test for trend across a group of two or more variables) and the method of cumulative divergence, which describes the extent to which each rank departs from the rank of the reference method; it takes into account both the fraction of divergent data and the extent of divergence for each analysed rank:

$$div_{cumulative}=\frac{AUC\{\sum_{i=1}^{n}\left(r_{i,ref}-r_{i,\;examined}\right)\}}{AUC\{\sum_{i=1}^{n}\left(r_{i,ref}-r_{i,ref-reversed}\right)\}},$$

where *n*, the total number of tested polyphenol-rich compounds, *r*_*i*,*ref*_, the rank of the *i*^*th*^ polyphenol-rich compound in the reference approach, *r*_*i*,*examined*_, the rank of the *i*^*th*^ polyphenol-rich compound in the examined approach, *r*_*i*,*ref-reversed*_, the rank of the *i*^*th*^ polyphenol-rich compound in the rank sequence reversed with regard to that of the reference approach, *div*_*cumulative*_, cumulative divergence and *AUC*, the area under the curve describing the function of $\sum_{i=1}^{n}\left(r_{i,ref}-r_{i,\;examined}\right)=f\left(rank\;sequence\right)$.

In addition, two graphical methods were used: a Bland-Altman plot (substantively identical with Tukey’s mean-difference plot) and a mountain plot, both of which are considered much better than a simple correlation analysis, as even a high correlation does not necessarily mean that the two methods have to be exchangeable [30].

To confirm that the evaluated associations were not observed by pure chance, the bootstrap-boosted Spearman’s rho rank correlation coefficient was used to assess associations among the measured variables (1000 iterations). Fisher’s exact test was used to compare the sensitivity of the cells through the assays or the assays through the cell lines. The statistical analyses were performed using the following software packages: *Statistica* v.12.5 and 13.1, *StatsDirect* v.3.0.182, *Resampling Stats Add-in for Excel* v.4, *GraphPad Prism* v.5.

## Results

### Cytotoxicity screening of polyphenol-rich compounds based on the type of an assay

#### Mitochondrial membrane potential

The representative dose-response curves obtained from the measurements of mitochondrial membrane potential, with the estimated total area and IC_50_, are shown in S1 Fig.

**Fig 2:**
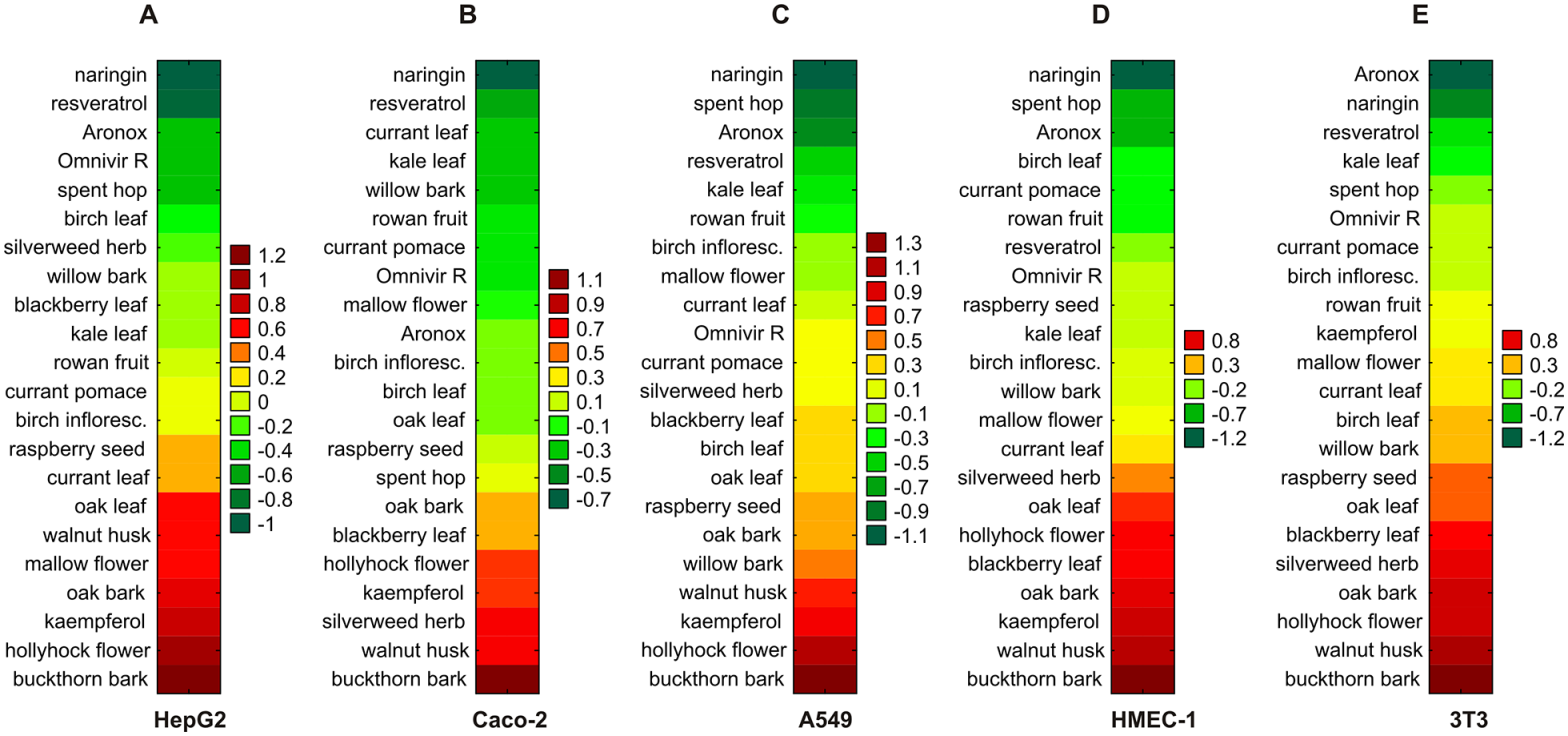
Prioritization of polyphenol-rich compounds with regard to their cytotoxicity in the measurements of mitochondrial activity. The toxicity of each polyphenol-rich compound was evaluated in five cell lines: HepG2 (A), Caco-2 (B), A549 (C), HMEC-1 (D), and 3T3 cells (E), based on the AUOC scores calculated for the mitochondrial membrane potential assay. The examined polyphenols were ranked in ascending order of toxicity towards the cells (increasing AUOC value). Naringin and kaempferol were used as negative and positive controls, respectively.

The modulators of mitochondrial membrane potential which had the lowest AUOC scores (the lowest cytotoxicity), were naringin, Aronox, resveratrol and the extracts from spent hop and currant leaf (Fig 2). Naringin demonstrated the lowest AUOC values in all cell lines but 3T3 cells, and hence it was one of the three least toxic agents with regard to mitochondrial potential among all the examined cell lines. Aronox was awarded one of the three lowest AUOC scores in four of five cell lines, i.e. all except Caco-2 cells. Similarly, resveratrol, but not the spent hop extract, demonstrated one of the three lowest AUOC scores for mitochondrial membrane potential in HepG2, Caco-2 and 3T3 cells. However, in A549 and HMEC-1 cells, the AUOC score values for the spent hop extract were the lowest of all polyphenols, excluding naringin. The extract from currant leaf was observed to be among the three least harmful agents to mitochondria in Caco-2 cells (Fig 2).

Keampferol and four extracts obtained from buckthorn bark, walnut husk, hollyhock flower and silverweed herb were among the three most cytotoxic compounds with regard to mitochondrial activity. The buckthorn bark extract had the highest AUOC scores in all the examined cell lines. Kaempferol and the extracts from walnut husk and hollyhock flower demonstrated the three highest AUOC scores in three of five cell lines. The silverweed herb extract was among the three most toxic to mitochondria in Caco-2 cells (Fig 2).

Regardless of cell line, the overall (cumulative) AUOC scores, calculated for all tested polyphenol-rich compounds in the mitochondrial membrane potential assay, are given in Table 2.

**Table 2:** Overall toxicity of the examined plant extracts summed up for all cell lines.

mitochondrial membrane potential		membrane permeability		nuclear area	
extract/compound	AUOC score	extract/compound	AUOC score	extract/compound	AUOC score
naringin	–5.16	naringin	–4.00	naringin	–3.30
Aronox	–3.40	resveratrol	–2.20	walnut husk	–2.30
resveratrol	–2.64	Aronox	–1.70	hollyhock flower	–2.10
spent hop	–2.32	spent hop	–1.10	buckthorn bark	–1.60
kale leaf	–1.47	rowan fruit	–0.80	birch infloresc.	–1.50
rowan fruit	–0.99	willow bark	–0.44	oak bark	–1.40
Omnivir R	–0.95	kale leaf	–0.37	spent hop	–1.40
currant pomace	–0.59	currant pomace	–0.08	oak leaf	–1.20
birch infloresc.	–0.37	birch leaf	0.08	kale leaf	–1.05
birch leaf	–0.33	currant leaf	0.49	currant leaf	–0.79
currant leaf	0.08	kaempferol	0.70	Aronox	–0.62
willow bark	0.17	raspberry seed	0.82	raspberry seed	–0.57
mallow flower	0.52	Omnivir R	1.07	Omnivir R	–0.38
raspberry seed	0.77	mallow flower	1.14	mallow flower	–0.12
silverweed herb	1.74	birch infloresc.	1.21	birch leaf	0.06
oak leaf	1.75	blackberry leaf	1.55	blackberry leaf	0.10
blackberry leaf	1.76	silverweed herb	1.82	silverweed herb	0.18
oak bark	2.86	oak leaf	2.20	currant pomace	0.50
kaempferol	2.88	oak bark	2.20	willow bark	0.60
walnut husk	3.90	hollyhock flower	2.60	rowan fruit	2.10
hollyhock flower	4.00	walnut husk	3.10	kaempferol	4.40
buckthorn bark	6.90	buckthorn bark	4.80	resveratrol	6.30

The estimates of plant extract toxicities were based on cumulative AUOC scores determined regardless of the cell line (summed AUOC score from individual AUOC scores obtained for each of five cell lines treated with a given extract/compound). Twenty-two polyphenols were ranked in ascending order of toxicity in each of the applied assays (increasing cumulative AUOC value).

#### Membrane integrity

**Fig 3:**
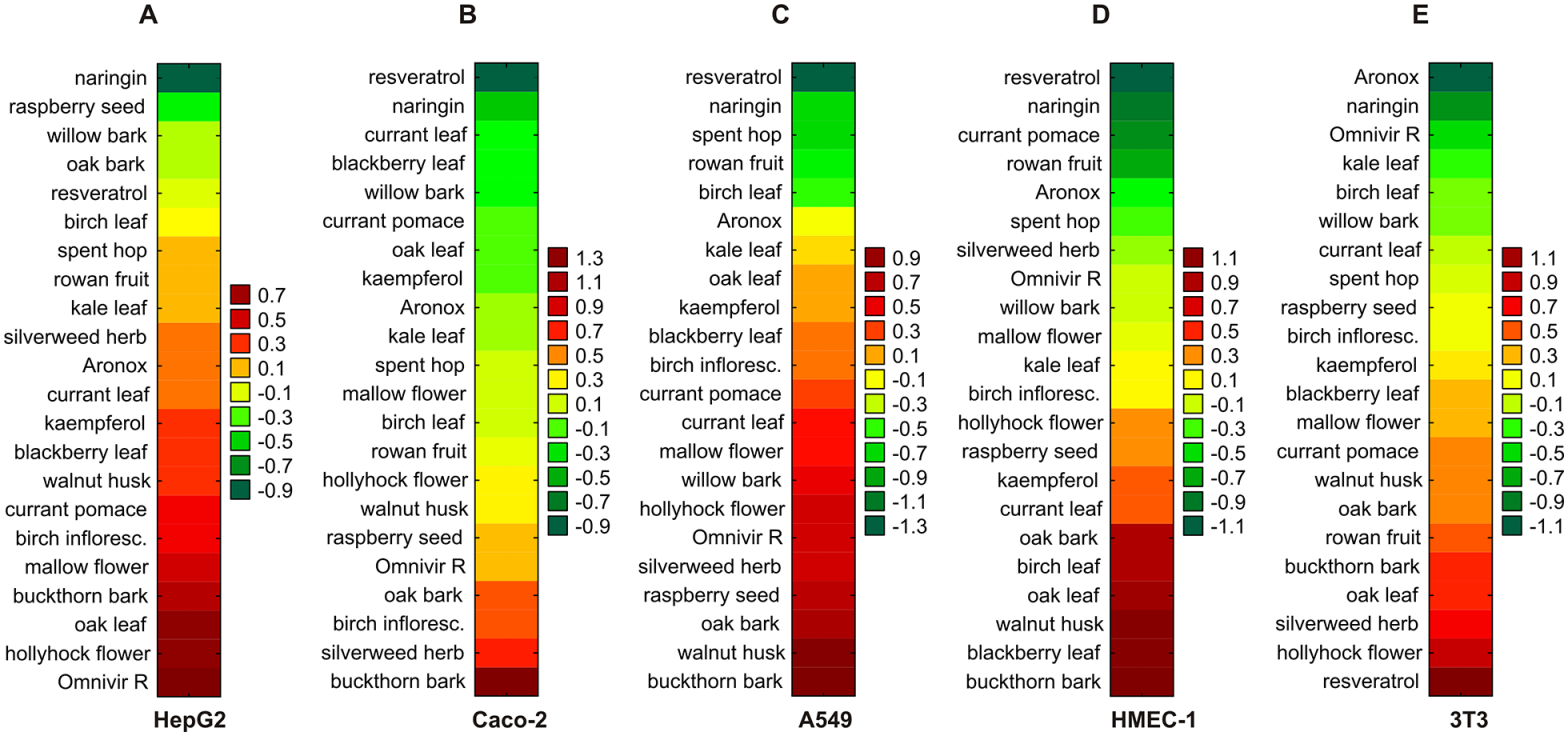
Hierarchical clustering of polyphenol-rich compounds for the estimation of plant extract toxicity in the analysis of cell membrane integrity. The toxicity of polyphenol-rich compounds was examined in five cell lines: HepG2 (A), Caco-2 (B), A549 (C), HMEC-1 (D), and 3T3 cells (E), based on the AUOC scores obtained in a test of cell membrane permeability. The examined polyphenols were ranked in ascending order of toxicity towards the cells (increasing AUOC value). Naringin and kaempferol served as negative and positive controls, respectively.

Regarding membrane integrity, the lowest AUOC scores against the analyzed cell lines were demonstrated by the polyphenols naringin, resveratrol, Aronox and Omnivir R, and the extracts of spent hop, currant leaf, currant pomace, raspberry seed and willow bark. No matter which cell line was used, naringin always demonstrated one of the lowest AUOC scores. Interestingly, resveratrol, which had the strongest protective effect against membrane disruption (the lowest AUOC scores) in Caco-2, A549 and HMEC-1 cells and exerted relatively low toxicity to HepG2 cell membranes, strongly enhanced membrane permeability in 3T3 fibroblasts, which was reflected by the highest AUOC score observed in 3T3 cells. Similarly, depending on cell line, Omnivir R exhibited either extremely protective (very low AUOC score in 3T3 cells) or extremely destructive effects (the highest AUOC score in HepG2 cells) on membrane permeability. The remaining six compounds were the least cytotoxic (with the lowest AUOC scores), but only with regard to single selected cell lines (Fig 3).

The extracts from buckthorn bark, walnut husk, hollyhock flower and silverweed herb were distinguished by their high toxicity regarding membrane integrity: they each demonstrated one of the three highest AUOC scores for cell permeability assay in two to three cell lines. The extracts from oak leaf (in HepG2 cells), birch inflorescences (in Caco-2 cells) and blackberry leaf (in HMEC-1 cells) were also among the agents exerting the most adverse effects on membrane integrity: each had one of the three highest AUOC values observed in single cell lines (Fig 3). The oak bark extract had one of the three highest AUOC values observed in A549 cells and relatively low AUOC scores in Caco-2, HMEC-1 and 3T3 cells. Also, worthy of note are the moderate AUOC score values noted for kaempferol in our experiments with all the examined cell lines.

Irrespective of the cell line used, the overall (cumulative) AUOC score values for all plant extracts calculated in the assay of membrane integrity are listed in Table 2.

#### Nuclear area

**Fig 4:**
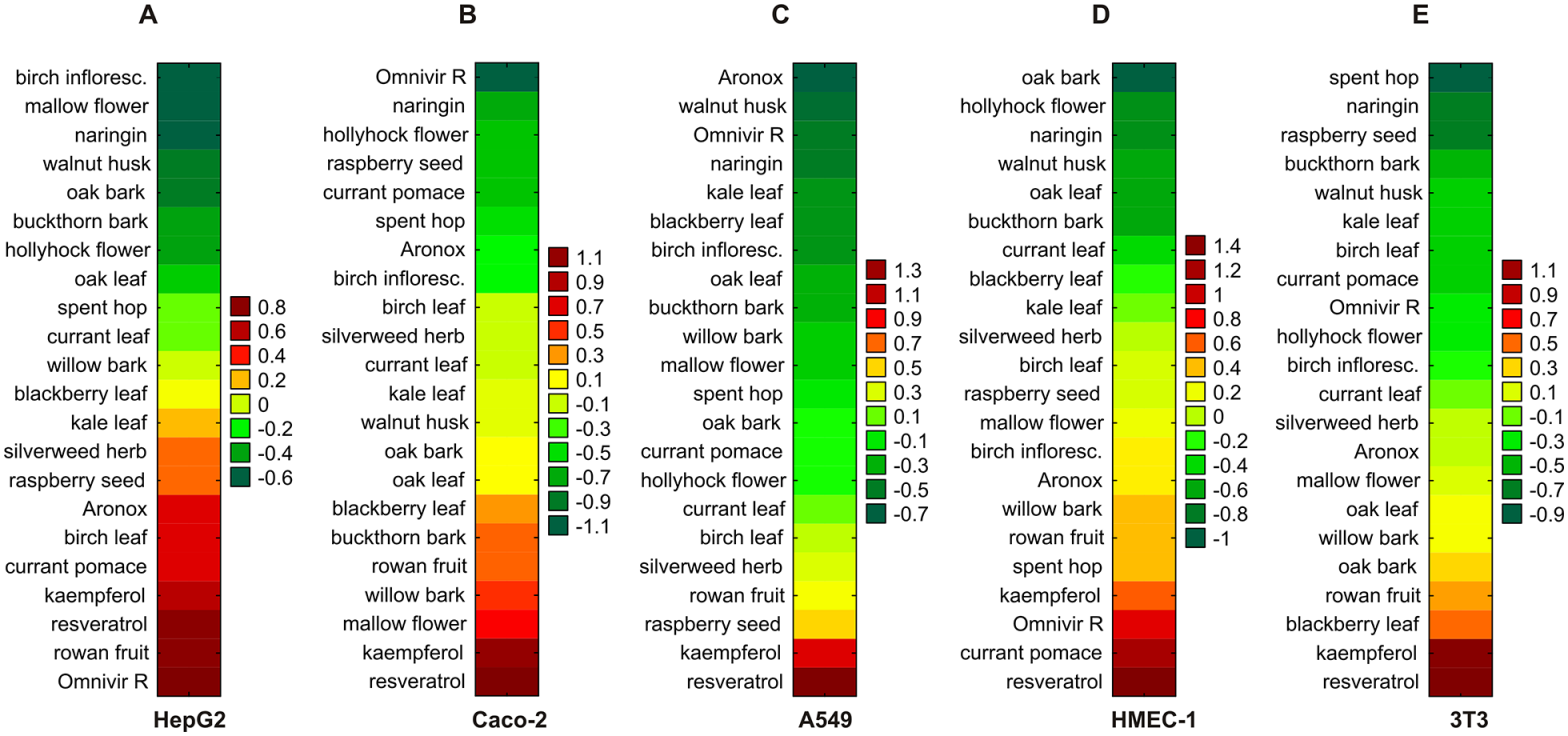
Prioritization of plant extracts and compounds in terms of nuclear morphology. The effect of polyphenol-rich compounds on nuclear area was assessed in five cell lines: HepG2 (A), Caco-2 (B), A549 (C), HMEC-1 (D), and 3T3 cells (E), based on the AUOC scores calculated in nuclear area assay. The examined polyphenols were ranked in ascending order of toxicity towards the cells (increasing AUOC value). Naringin and kaempferol were used as negative and positive controls, respectively.

Naringin, Omnivir R, Aronox and the extracts from hollyhock flower, spent hop, oak bark, raspberry seed, mallow flower, birch inflorescence and walnut husk were some of the least toxic compounds in nuclear area measurements. Naringin gave one of the three lowest AUOC scores in all tested cell lines apart from A549 epithelial cells. Hollyhock flower extract demonstrated low AUOC scores against Caco-2 and HMEC-1 cells. Omnivir R had one of the three lowest AUOC scores in Caco-2 and A549 cells, but at the same time, it had one of the three highest AUOC scores in HepG2 and HMEC-1 cells. Likewise, the extracts from raspberry seed and mallow flower, which had one of the three lowest AUOC scores, respectively in 3T3 and HepG2 cells, were within the three most cytotoxic agents in A549 and Caco-2 cells, respectively. Each of the remaining five polyphenols (Aronox and the extracts from spent hop, oak bark, birch inflorescence and walnut husk) was ranked within the three least cytotoxic polyphenols, with the lowest AUOC scores found only in one of the five cell lines examined (Fig 4).

Resveratrol and kaempferol had one of the highest AUOC scores concerning the nuclear area: they remained among the three most toxic polyphenols in this regard (Fig 4). Resveratrol had the highest AUOC scores in all cell lines but HepG2 cells, whereas kaempferol showed one of the four highest AUOC scores in all examined cell lines. Blackberry leaf extract was in the top three AUOC scores for 3T3 cells and currant pomace extract in HMEC-1 cells. Furthermore, the extract from rowan fruit exerted relatively high toxicity against the examined cell lines, having one of the six highest AUOC values (Fig 4).

The toxicity of all polyphenol-rich compounds expressed by the values of overall (cumulative) AUOC scores obtained in the measurements of nuclear area are presented in Table 2.

### Cytotoxicity screening of polyphenol-rich compounds in relation to the cell line

#### HepG2 human hepatocytes

Naringin, Aronox, resveratrol, and the extracts from birch inflorescence, mallow flower, raspberry seed, and willow bark were the least toxic agents in HepG2 cells (Figs 2A, 3A and 4A). However, only naringin was one of the three least toxic compounds in all the assays. Aronox, and the extracts from birch inflorescence raspberry seed and willow bark were characterized by the lowest AUOC scores only in one assay. Resveratrol, the rowan fruit extract (which exhibited low to moderate toxicity to mitochondrial activity and membrane integrity) and Omnivir R represented the most toxic compounds in the study of nuclear area. The extract from mallow flower presented the opposite characteristics to that of resveratrol in the sense that although the mallow flower extract induced relatively small changes in nuclear area, it had relatively high AUOC scores in other assays. Omnivir R, kaempferol, and the extracts from hollyhock flower, buckthorn bark, and oak leaf were characterized by high AUOC scores in two out of three assays (Figs 2A, 3A and 4A).

The values of overall (cumulative) AUOC scores calculated for all plant polyphenol-rich compounds used in HepG2 cells, across all three assays are given in Table 3.

**Table 3:** Overall cumulative toxicity scores of the examined plant extracts for all assays.

HepG2		Caco-2		A549		HMEC-1		3T3	
extract/compound	AUOC score	extract/compound	AUOC score	extract/compound	AUOC score	extract/compound	AUOC score	extract/compound	AUOC score
naringin	–2.63	Naringin	–1.94	naringin	–2.43	naringin	–2.90	naringin	–2.56
spent hop	–0.62	Omnivir R	–0.99	spent hop	–1.86	Aronox	–0.97	Aronox	–2.46
willow bark	–0.50	currant pomace	–0.96	Aronox	–1.70	rowan fruit	–0.86	spent hop	–1.29
resveratrol	–0.38	currant leaf	–0.76	kale leaf	–0.97	spent hop	–0.72	kale leaf	–1.19
birch infloresc.	–0.29	Aronox	–0.67	rowan fruit	–0.71	kale leaf	–0.25	Omnivir R	–1.15
oak bark	–0.12	kale leaf	–0.49	birch infloresc.	–0.48	currant pomace	–0.17	birch leaf	–0.49
raspberry seed	–0.01	spent hop	–0.32	resveratrol	–0.34	currant leaf	0.00	birch infloresc.	–0.34
kale leaf	0.02	raspberry seed	–0.28	blackberry leaf	–0.16	silverweed herb	0.05	raspberry seed	–0.27
birch leaf	0.04	resveratrol	–0.14	birch leaf	–0.11	hollyhock flower	0.09	currant pomace	–0.25
Aronox	0.07	birch leaf	–0.14	oak leaf	–0.04	raspberry seed	0.15	currant leaf	–0.08
silverweed herb	0.13	willow bark	–0.13	mallow flower	0.03	resveratrol	0.16	willow bark	0.20
blackberry leaf	0.20	oak leaf	–0.13	Omnivir R	0.17	willow bark	0.17	mallow flower	0.46
walnut husk	0.30	hollyhock flower	0.13	currant leaf	0.30	mallow flower	0.24	rowan fruit	0.87
currant leaf	0.32	birch infloresc.	0.13	currant pomace	0.39	birch infloresc.	0.28	walnut husk	0.95
mallow flower	0.38	rowan fruit	0.20	willow bark	0.59	oak bark	0.46	oak leaf	1.08
rowan fruit	0.75	blackberry leaf	0.39	silverweed herb	0.95	birch leaf	0.51	hollyhock flower	1.34
currant pomace	0.81	mallow flower	0.43	oak bark	1.01	Omnivir R	0.53	blackberry leaf	1.38
oak leaf	0.89	oak bark	0.85	walnut husk	1.06	oak leaf	0.93	silverweed herb	1.43
Omnivir R	1.18	walnut husk	0.92	raspberry seed	1.44	walnut husk	1.40	buckthorn bark	1.46
buckthorn bark	1.25	silverweed herb	1.17	hollyhock flower	1.55	blackberry leaf	1.59	oak bark	1.47
hollyhock flower	1.33	kaempferol	1.50	kaempferol	1.75	kaempferol	1.77	kaempferol	1.49
kaempferol	1.48	buckthorn bark	2.96	buckthorn bark	2.27	buckthorn bark	2.05	resveratrol	2.20

The estimates of plant extract toxicities were based on cumulative AUOC score determined regardless of the assay (summed AUOC score from individual AUOC scores obtained for each of three assays with a given extract/compound). Twenty-two polyphenols were ranked in ascending order of toxicity in each examined cell line (increasing cumulative AUOC value).

#### Caco-2 intestinal cells

The three least toxic agents, characterized by the lowest AUOC scores in Caco-2 cells, included naringin, resveratrol, Omnivir R and the extracts from currant leaf and hollyhock flower, irrespective of assay (Figs 2B, 3B and 4B). According to the AUOC score, naringin was one of the two least toxic polyphenols in all assays. Resveratrol and the extract from currant leaf each had one of the three lowest AUOC scores in two assays: mitochondrial membrane permeability and cell membrane permeability. In addition to naringin, Omnivir R and the hollyhock flower extract were the least toxic polyphenols in the analysis of nuclear area (Figs 2B, 3B and 4B).

In the study of mitochondrial membrane potential and membrane permeability, the extracts from buckthorn bark and silverweed herb were distinguished by the highest toxicity, showing the highest AUOC scores in these methods. High AUOC scores were also obtained for kaempferol in the measurements of mitochondrial membrane potential and nuclear area. Resveratrol, as well as the extracts from walnut husk, mallow flower, and birch inflorescence were in the group of the three highest AUOC scores found in only one used assay (Figs 2B, 3B and 4B).

The values of overall (cumulative) AUOC scores calculated for all plant extracts in the experiments with Caco-2 intestinal cells, across all the applied assays, are presented in Table 3.

#### A549 epithelial cells

Generally, naringin, Aronox, Omnivir R, resveratrol, and the extracts from spent hop and walnut husk were among the three least toxic polyphenol-rich compounds in the A549 cell line (Figs 2C, 3C and 4C). On the other hand, naringin, Aronox, and the kale leaf extract were shown to have relatively low AUOC scores in all assays. Resveratrol and the spent hop extract exerted low toxicity in two assays (mitochondrial membrane potential and membrane integrity), while Omnivir R and the walnut husk extract were characterized by the three lowest AUOC scores only in one assay (nuclear area). At the same time, high toxicity was exhibited by resveratrol (nuclear area) and the walnut husk extract (membrane integrity and mitochondrial membrane potential). Otherwise, the most toxic polyphenol-rich compounds in the experiments with A549 cells appeared to be kaempferol and the extracts from buckthorn bark, hollyhock flower, raspberry seed and oak bark, which demonstrated relatively high AUOC scores in two out of three methods (Figs 2C, 3C and 4C).

The overall (cumulative) AUOC score values obtained in the analysis of toxicological effects of the examined polyphenol-rich compounds using A549 epithelial cells are listed in Table 3.

#### Human microvascular endothelial cells (HMEC-1)

In all assays performed in HMEC-1 cells, naringin had one of the three lowest AUOC scores. Aronox, resveratrol, as well as the extracts from spent hop, currant pomace and rowan fruit had relatively low AUOC scores in the experiments with mitochondrial potential and cell membrane permeability, but, at the same time, they exhibited high scores when nuclear area was measured. In turn, the extracts from hollyhock flower and oak bark, which were the two least toxic extracts in the measurements of nuclear area, had relatively high AUOC scores in the remaining assays (Figs 2D, 3D and 4D). The extracts from buckthorn bark, walnut husk, blackberry leaf and oak leaf were found to have high toxicity by the mitochondrial activity and membrane integrity assays. The three most toxic polyphenol-rich compounds in the study of nuclear area were resveratrol, the extract from currant pomace and Omnivir R. In addition, kaempferol was distinguished by high AUOC scores in all three methods.

All overall (cumulative) AUOC scores calculated for the examined plant extracts against human microvascular endothelial cells, irrespective of the method used, are shown in Table 3.

#### 3T3 mouse fibroblasts

When the 3T3 cell line was used, some of the lowest AUOC scores were achieved by naringin (in all three methods) and Aronox (in two out of three methods) (Figs 2E, 3E and 4E), followed by Omnivir R and the extracts from spent hop and kale leaf. In two out of three assays, resveratrol and the extracts from hollyhock flower, buckthorn bark, silverweed herb, and blackberry leaf showed relatively high toxicity. Kaempferol, after resveratrol, was the most toxic polyphenol in the nuclear area test. The oak bark extract had relatively high AUOC scores in all three assays (Figs 2E, 3E and 4E).

The overall (cumulative) AUOC score values, obtained for the plant extracts in the toxicity analysis with 3T3 mouse fibroblasts, are given in Table 3.

### Comparison of cell lines and methods

In the mitochondrial membrane potential assay, a significant positive correlation between cell lines was revealed in ten out of ten possible pair combinations of cell lines (Table 4). Regarding the membrane permeability test, significant correlation coefficients regarding AUOC scores were found only in two out of ten possible pair combinations of cell lines: A549 and Caco-2 (*P* = 0.007), and A549 and HMEC-1 (*P* = 0.031). When nuclear area was measured, significant correlation coefficients were also observed in two out of ten possible pair combinations, namely between 3T3 and Caco-2 (*P* = 0.003) and HepG2 and HMEC-1 (*P* = 0.003) (Table 4).

**Table 4:** Associations between cell lines used for estimating toxicity of polyphenol-rich compounds.

cell line	mitochondrial membrane potential				membrane permeability				nuclear area			
	Caco-2	A549	HMEC-1	3T3	Caco-2	A549	HMEC-1	3T3	Caco-2	A549	HMEC-1	3T3
HepG2	0.533	0.713	0.760	0.667	0.273	0.346	0.307	0.264	0.026	0.387	0.656	0.280
	*P* = 0.021	*P* < 0.001	*P* < 0.0001	*P* = 0.003	NS	NS	NS	NS	NS	NS	*P* = 0.003	NS
Caco-2		0.656	0.627	0.712		0.607	0.294	0.224		0.265	0.125	0.668
		*P* = 0.003	*P* = 0.005	*P* < 0.001		*P* = 0.007	NS	NS		NS	NS	*P* = 0.003
A549			0.764	0.838			0.498	0.247			0.278	0.285
			*P* < 0.001	*P* < 0.0001			*P* = 0.031	NS			NS	NS
HMEC-1				0.785				0.150				0.237
				*P* < 0.0001				NS				NS

Data are presented as bootstrap-boosted Spearman's *correlation coefficients* (*rho*) with *P*-*values*.

In all cell lines significant correlations were found between assays: one out of three associations was found in Caco-2, A549 cells, 3T3 and HepG2 cells and three associations were seen in HMEC-1 cells (Table 5).

**Table 5:** Associations between assays used for estimating toxicity of polyphenol-rich compounds.

**Assay**	membrane permeability	nuclear area
	**HepG2**	
mitochondrial membrane potential	0.449	-0.384
	*P* = 0.054	NS
membrane permeability		-0.139
		NS
	**Caco-2**	
mitochondrial membrane potential	0.569	0.015
	*P* = 0.013	NS
membrane permeability		-0.228
		NS
	**A549**	
mitochondrial membrane potential	0.699	0.114
	*P* = 0.002	NS
membrane permeability		-0.157
		NS
	**HMEC-1**	
mitochondrial membrane potential	0.692	-0.485
	*P* = 0.002	*P* = 0.036
membrane permeability		-0.580
		*P* = 0.011
	**3T3**	
mitochondrial membrane potential	0.506	0.004
	*P* = 0.029	NS
membrane permeability		0.303
		NS

Data are presented as bootstrap-boosted Spearman's *correlation coefficients* (*rho*) with *P*-*values*.

Susceptibility of cell lines to the examined polyphenol-rich compounds, estimated by the number of mean AUOC scores above 0, varied depending on the method (Table 6). In the mitochondrial membrane potential assay, the least sensitive cell line to the polyphenol-rich compounds was Caco-2 and the most sensitive was 3T3. In the study of membrane permeability, the Caco-2 cell line was the least susceptible and HepG2 the most. For the nuclear area test, A549 was the least sensitive cell line and HMEC-1 the most. When using this approach, we revealed no association between the cell line and sensitivity.

**Table 6:** The numbers of plant compounds with positive AUOC score evaluated with various assays for different cell lines.

	mitochondrial membrane potential	membrane permeability	nuclear area
	(n = 22)	(n = 22)	(n = 22)
**HepG2**	11	16	11
**Caco-2**	9	12	9
**A549**	13	15	7
**HMEC-1**	10	12	12
**3T3**	14	14	8

The polyphenol-rich compounds showing AUOC>0, referred to as ‘cytotoxic’, were established to evaluate the sensitivity of three assays in high content analysis of toxicity with regard to five different cell lines.

The sensitivity of methods was assessed according to the number of mean AUOC scores exceeding 0 in relation to the cell line used (Table 6). The cell membrane permeability assay had the highest numbers of positive AUOC cases in HepG2, Caco-2 and A549 cells. The number of positive AUOC cases in the membrane integrity assay was greater than or equal to mitochondrial membrane potential assay in the HMEC-1 and 3T3 cell lines. The nuclear area assay had the lowest number of positive AUOC cases in experiments with A549 and 3T3 cells, and less positive AUOC cases than the membrane integrity assay in HepG2, Caco-2, A549, and 3T3 cells. When using this approach, we revealed no association between the assay and sensitivity.

### Assessment of polyphenol-rich compound toxicity in comparison to control phytochemicals

Based on the cumulative AUOC scores calculated within each line across the three assays (mitochondrial potential, membrane integrity and nuclear area) the toxicity of the examined polyphenol-rich compounds were compared with that of the control phytochemicals, naringin and kaempferol.

In 80% of cases (in 80 out of 100 comparisons; 20 comparisons per cell line, excluding kaempferol), the toxicity of naringin remained significantly lower than that observed in other polyphenol-rich compounds (for various comparisons: *P*<0.05 or lower). Spent hop extract (in HepG2, Caco-2, A549, and 3T3), kale leaf extract (in Caco-2, A549 cells, and 3T3), willow bark extract (in HepG2 and Caco-2), currant pomace extract (Caco-2 and 3T3), blackberry leaf extract (in Caco-2 and A549), birch inflorescence extract (in A549 and 3T3), rowan fruit extract (in Caco-2 and HMEC-1), Aronox (in 3T3), currant leaf extract (in Caco-2) and oak leaf extract (in Caco-2) demonstrated insignificantly lower toxicity than that of naringin.

Considering all the tested cell lines, the toxicity of kaempferol was not different from that of the extracts from walnut husk, mallow flower and silverweed herb. In four out of five cell lines, the AUOC scores of kaempferol and the extracts from oak bark, blackberry leaf, oak leaf, birch leaf, and hollyhock flower were not significantly different. The differences were found in HMEC-1 cells (oak bark, *P*<0.035), in A549 cells (blackberry leaf and oak leaf, *P*<0.005 and *P*<0.007), and in Caco-2 cells (birch leaf and hollyhock flower, *P*<0.015 and *P*<0.03).

In Caco-2 and HMEC-1 cells the toxicity of kaempferol was significantly higher than that of resveratrol (*P* = 0.04 or lower), whereas in Caco-2 and A549 it was significantly higher than for willow bark (*P*<0.03 or less), currant pomace (*P*<0.04 or less), currant leaf (*P*<0.015 or less) and rowan fruit (*P*<0.005 or less). In contrast, kaempferol had significantly lower toxicity than the buckthorn bark extract in Caco-2 and A549 cells (*P*<0.006 or less). In three out of five cell lines, kaempferol was considerably more toxic than Omnivir R (in Caco-2, HMEC-1 and 3T3, *P*<0.05 or less), spent hop extract (in A549, HMEC-1 and 3T3, *P* = 0.02 or less), and raspberry seed extract (in Caco-2, HMEC-1 and 3T3, *P*<0.03 or less). In all cell lines, Aronox had significantly lower scores than kaempferol, except for HepG2 (*P*<0.006 or less). In all cell lines, the differences between the toxicity of kaempferol and the examined polyphenols remained statistically significant for naringin (*P*<0.025 or less) and the extracts from kale leaf (*P*<0.015 or less) and birch inflorescence (*P*<0.04 or less).

The list of the five least toxic and the five most toxic polyphenol-rich compounds in the study is presented in Table 7. The chosen polyphenols were characterized by the lowest or the highest global AUOC scores, summed across the assays and across the cell lines.

**Table 7:** The five least and the five most toxic polyphenol-rich compounds monitored for five cell lines.

	HepG2	Caco-2	A549	HMEC-1	3T3	global AUOC score
**the least toxic**						
naringin						-12.46
Aronox	*P*<0.0025	*P*<0.0001	*P*<0.005	*P*<0.0001	NS	-5.72
spent hop	NS	NS	NS	*P*<0.0002	NS	-4.81
kale leaf	*P* = 0.01	NS	NS	*P*<0.035	NS	-2.89
birch infloresc.	*P*<0.025	*P*<0.05	NS	*P* = 0.011	NS	-0.69
Omnivir R	*P*<0.0001	*P*<0.004	*P*<0.0001	*P*<0.0001	*P*<0.0035	-0.26
**the most toxic**						
kaempferol						7.99
buckthorn bark	NS	*P*<0.006	*P*<0.003	NS	NS	9.99
walnut husk	NS	NS	NS	NS	NS	4.63
hollyhock flower	NS	*P*<0.03	NS	NS	NS	4.44
silverweed herb	NS	NS	NS	NS	NS	3.74
oak bark	NS	NS	NS	*P*<0.035	NS	3.67

The selection of polyphenol-rich compounds was based on the values of global AUOC score (summed over three assays) to give five polyphenolic compounds with the lowest AUOC score and five polyphenolic compounds with the highest. The differences between the examined extracts and the reference compounds (naringin and kaempferol) were determined using one-way ANOVA and the Fisher's least significant difference (LSD) test. The *P* values presented in the table for the least toxic polyphenols concern the statistical differences revealed between Aronox, Omnivir R, the extracts from spent hop, kale leaf and a negative control—naringin. The *P* values presented in the table for the most toxic polyphenols concern the statistical differences found between the extracts from buckthorn bark, walnut husk, hollyhock flower, silverweed herb, oak bark and a positive control—kaempferol. For details, please see Materials & Methods.

When we applied block analysis to adjust for the effects of the cell line and the assay, we demonstrated that while the first showed merely a negligible impact (*F*_block ANOVA: cell line-adjusted_ = 10.85 *vs*. *F*_one-way ANOVA: non-adjusted_ = 10.79; for both *P*<<0.0001), the second resulted in over twofold of *F* statistics (*F*_block ANOVA: assay-adjusted_ = 26.42 *vs*. *F*_block ANOVA: non-adjusted_ = 10.79; for both *P*<<0.0001). When adjusted at once for two grouping co-variates, the assay and the cell line, the resultant *F*_block ANOVA_ statistics did not improve considerably (*F*_block ANOVA: assay/cell line-adjusted_ = 26.83; *P*<<0.0001). Again, the toxicity of naringin remained significantly lower than other tested polyphenol-rich compounds (*P*<<0.0001). Aronox appeared significantly less toxic than nearly all other polyphenol-rich compounds but the extracts from birch inflorescence, spent hop and kale leaf (*P*<0.05 or less). Kaempferol and buckthorn bark were the most toxic compounds with regard to all the remaining polyphenol-rich compounds.

The least toxic compounds, based on a double adjustment for both the assay and the cell line, were (naringin, Aronox), the extracts from spent hop, kale leaf, birch inflorescence and currant pomace. The most toxic were the extract from buckthorn bark, (kaempferol), the extracts from hollyhock flower, walnut husk, silverweed herb and blackberry leaf. Overall, the differences between the least toxic compounds and the negative control (naringin), evaluated by the *post hoc* multiple comparisons Fisher’s LSD test, following the block ANOVA adjusted for assay/cell line, were: Aronox (*P*<0.0001), spent hop extract (*P*<0.0001), kale leaf extract (*P*<0.0001), birch inflorescence extract (*P*<0.0001), currant pomace extract (*P*<0.0001) and rowan fruit extract (*P*<0.0001). The differences between the most toxic compounds and the positive control (kaempferol) were as follows: buckthorn bark extract (*NS*), hollyhock flower extract (*P*<0.05), walnut husk extract (*P*<0.05), silverweed herb extract (P<0.02) and blackberry leaf extract (*P*<0.01).

**Fig 5:**
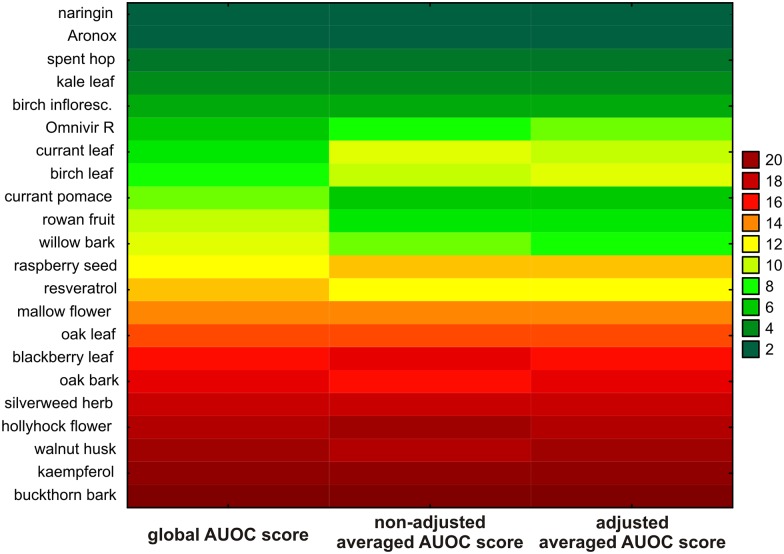
The toxicity rank of polyphenol-rich compounds according to global AUOC-based normal score, non-adjusted averaged AUOC-based normal score and double-adjusted averaged AUOC-based normal score. The examined polyphenol-rich compounds were ranked in ascending order of toxicity through the cell lines and assays (increasing global AUOC-based normal scores).

Finally, three different approaches to ranking cytotoxicity were compared: (*1*) global normalized AUOC scores (from now on referred to as ‘global AUOC’), (*2*) non-adjusted averaged individual normal scores of AUOC (from now on referred to as ‘non-adjusted normalized AUOC’), and (*3*) averaged individual normal scores of AUOC adjusted for both the assay and the cell line (from now on referred to as ‘double-adjusted normalized AUOC’). All employed statistical and graphical techniques clearly indicated considerable agreement between three approaches (Fig 5). No significant differences were found between the sequences of ranks assigned to the toxicities of polyphenol-rich compounds evaluated with the use of the three approaches (one-tailed *P* = 0.50 by Cuzick’s test). The agreement between the approaches, given by AC1 Gwet’s method, was 42.46% and 42.86% for comparing ‘global AUOC’ with ‘non-adjusted normalized AUOC’ and ‘global AUOC’ with ‘double-adjusted normalized AUOC’, respectively, and 71.43% for comparing ‘non-adjusted’ with ‘double-adjusted normalized AUOC’. Likewise, the cumulative divergence values for these comparisons were 9.95%, 9.95% and 6.33%, clearly pointing to better agreement between non-adjusted and double-adjusted normalized AUOC than between global AUOC and normalized averaged individual AUOC. Also, the graphical methods, i.e. the Bland-Altman plots (S2 Fig) and mountain plots (S3 Fig), confirmed a high degree of consistency between the employed approaches.

## Discussion

The present study uses the HCS assay to prioritize twenty-two polyphenol-rich compounds through five established cell lines, according to their *in vitro* toxicity profile determined by total area under and over the dose-response curves obtained in the measurements of mitochondrial membrane potential, cell membrane permeability and nuclear area. The cytotoxicity analysis involved seventeen crude plant extracts, two commercial plant extracts (Aronox and Omnivir R), as well as three well-known phytochemicals regularly consumed by humans, like resveratrol, naringin (negative control), and kaempferol (positive control).

The majority of the examined compounds (the exception can be naringin) demonstrated different toxicity profiles between the methods. Some polyphenol-rich compounds that were assessed as very safe (the spent hop extract, Aronox, resveratrol) or quite safe (kale leaf, rowan fruit) with regard to their cytotoxic effects on mitochondrial membrane potential and plasma membrane integrity were found to be harmful in the analysis of nuclear area (in particular, resveratrol). On the other hand, another group of extracts, like those from buckthorn bark, walnut husk, hollyhock flower, oak bark, oak leaf, silverweed herb, and blackberry leaf, were highly cytotoxic with regard to mitochondrial membrane potential and cell membrane permeability, but quite inert in mediating nuclear morphology abnormalities. This observation was most pronounced when the cumulative effect of polyphenol-rich compound action was analyzed (the cumulative AUOC score calculated for all cell lines) (Table 2). Furthermore, kaempferol exerted strong cytotoxicity both on the level of mitochondria and nuclei, but unexpectedly, only moderately affected plasma membrane permeability. The above data identifies a clear need to examine the toxicity of biologically-active compounds such as plant extracts with caution. Considering their chemical heterogeneity and potential multidirectional effects, a toxicological characteristics of plant extracts should be supported by multiparametric analyses in order to unveil any detrimental cellular effects.

### Utility of approach

The paper discusses the use of normalized AUOC value, obtained by the integrating of the area under and over the dose-response curve (fluorescence *vs*. polyphenol concentration), as an approach useful to assess toxicity of plant compounds. This approach allows cell response to polyphenol-rich compounds tested to be studied at only a few concentrations (five doses within the range of 5–50 μg/ml). Under such conditions, the AUOC was always possible to be calculated (Figs 2–4). However, any attempt to estimate the AC_50_ value from raw data was difficult or just impossible, as in many cases, the data did not present an apparent sigmoid curve-like dependence. As a consequence, we were not be able to reliably compare AUOC and the AC_50_ value reflecting the toxicity. However, to overcome this problem, the data obtained in the independent colorimetric MTT assay was analyzed for as many as 12 concentrations of selected polyphenolic compound. This data originated from the study of anti-proliferative effects of two commercial preparations of resveratrol on human endothelial cells [28]. In practice, those preparations demonstrated quite different IC_50_ values (the averaged IC_50_ were respectively, about 40 μg/ml for freshly purchased preparation and 25 μg/ml for the older preparation). For the purposes of the present study, this resveratrol data was used to compare the calculated values of IC_50_ with those of the AUOC; a significant negative correlation was found between the numerical values of IC_50_ and total areas (AUOC) (*R*_S_ = −0.409, n = 32, *P* = 0.0101) (S2 Table), indicating that the less toxic plant compounds are characterized by the lower AUOC values. Of note, in the present work, the least toxic compounds had the lowest AUOC score and the compounds with the highest toxicity had the highest AUOC scores.

Taken together, our results may indicate that our approach for rank ordering compound toxicity has great potential, and suggest that AUOC score may be used as an alternative measure of toxicity to the standard AC_50_ value [31]. The presented methodology appears to be a suitable and effective tool that makes it possible to evaluate and rank cytotoxicity even under circumstances that preclude classical measures like AC_50_. Although the problems with interpreting data from toxicological studies are not so rare, the measures of toxicity other than AC_50_ are seldom reported. Recently, Martin et al. proposed the use of the so called Z-factor, the measure of a statistical effect size in a high-content screening procedure for the identification of cytotoxic compounds based on cell morphology and cell proliferation markers [13]. Furthermore, in HCS assay employed for the prediction of human drug-induced liver injury (DILI), drugs with known clinical hepatotoxicity were hierarchically clustered for toxicity based on the IC_50_ value and lowest efficacious concentration (LEC) [15]. It should be stressed herein that the use of standard sigmoidal curves to obtain the IC_50_ values is inappropriate in a number of compounds, because they can exert markedly differentiated effects at higher concentrations, the initial cell response is small, or the response consists of multiple phases. In the majority of these cases, the authors attempted to establish a toxicity profile of a given compound in an arbitrary manner, either using other IC values or analyzing only a part of the curve [32]. For compounds which produce dual effects, a modified version of the Brain and Cousens curve has been used to model the response [4]. Also, a role is played by the hormesis phenomenon (biphasic response), which is commonly manifested in both biological and toxicological settings with a frequency of about 40% [33–36]. Being a protective mechanism derived from metabolic adaptation to environmental stresses, hormesis has been shown to influence cell number, mitochondrial activity, nuclear area, intracellular calcium level, membrane permeability and other activities [7,37,38]. For example, hormetic effects have been observed in response to endogenous agonists, drugs and natural compounds, like daidzein, genistein, curcumin, naringin or resveratrol [36]. It is important to note that the present study compares a few slightly different calculus scenarios used for estimating the measures of cytotoxicity. Apparently, regardless of the scenario used (global normalized AUOC or averaged individual normalized AUOC), similar resultant rankings were obtained for estimated cytotoxicity. Most importantly, very good agreement was found with the use of statistical and graphical methods between various calculus scenarios.

### Assays and cell lines

High content screening technology enabled the simultaneous measurement of a few cell health indicators associated with mitochondrial function, plasma membrane integrity and nuclear morphology. Mitochondrial dysfunction is a well-documented *in vivo* primary mechanism of toxicity in humans that can account for liver, kidney and neuronal effects [39]. Changes in plasma membrane permeability, leading to the loss of plasma membrane integrity, may indicate an adverse or an adaptive response. Highly vulnerable to *in vivo* mechanically-induced plasma membrane disruption are cardiac and skeletal muscle cells, epithelial cells, and endothelial cells [40]. By analysing nuclear morphometric features such as nuclear size, it is possible to provide an insight into the dynamics of cell growth and death, and the development and progression of cancer [41,42].

To investigate the adverse biological effects of polyphenol-rich compounds, cell lines were chosen which are well characterized, easy to culture and have been already preferably used in published cytotoxicity measurements. Such selection criteria have been already proposed in the literature [43]. In addition, the study uses the murine 3T3 cell line, which is probably the single most widely-used cell line for *in vitro* toxicity assays: it has been validated for multiple compounds and approved by ICCVAM for Neutral Red Uptake cytotoxicity assay (https://ntp.niehs.nih.gov/iccvam/methods/acutetox/invidocs/phiiiprot/3t3phiii.pdf).

Cell lines representing different phenotypes were chosen: HepG2, representing a phenotype of hepatocytes, and Caco-2, representing the intestinal epithelium, which is frequently employed in ADME-Tox studies and is directly related to organs that are critically important for the response to xenobiotics entering a body *via* oral route. These cell lines were supplemented by two additional cell lines, A549 and HMEC-1, which are derived respectively from lung and skin, and represent alternative routes of exposure to xenobiotics. However, it has to be stressed that polyphenol-rich compounds need to be screened primarily through metabolically competent cells, as their chemically-reactive metabolites, produced in the course of metabolic processes, account for > 50% human liver injury [44]. Apart from primary human hepatocytes, hepatoma cells (e.g. HepG2 cells) and recently developed human upcyte hepatocytes offer suitable properties for use in toxicological assessments during drug development [45].

In the study of mitochondrial membrane potential, the degree of toxicity of each tested polyphenol-rich compound was found not to differ between the examined cell lines: significant positive correlations were observed between the AUOC scores of various cell lines in ten out of ten possible cell pair combinations (Table 4). In contrast, when membrane permeability was evaluated, significant correlations were only observed between A549 and Caco-2 or HMEC cells. In the nuclear area analysis, significant correlation coefficients were found in two out of ten possible cell pair combinations, including the associations between HepG2 and HMEC-1 and between Caco-2 and 3T3 cells. Altogether, these findings suggest that the examined polyphenols may, to some extent, exert similar effects in non-hepatic cells to those observed in hepatic cells. Hence, metabolically competent HepG2 cells may not be the only suitable cell line for predicting the toxicity potential of plant compounds with regard to the employed assay of investigation. Although none of the applied non-hepatic cells was as effective as HepG2 in the evaluation of cell membrane integrity following the treatment with polyphenol-rich compounds, endothelial cells (HMEC-1) were found to effectively substitute HepG2 cells in the assessment of mitochondrial activity and nuclear morphology (Table 5). Similarly, Lieggi et al. reported an apparent equivalence between human HepG2 and human T-lymphocytes for the identification of cytotoxic agents, when comparing those cell lines for the assessment of anti-cancer drug cytotoxicity [37]. In addition, quite recently canine peripheral blood lymphocytes have also been demonstrated to be suitable in the detection and monitoring of *in vivo* drug toxicity by high-content analysis [7].

The analysis of cumulative AUOC scores calculated for three assays together allowed an assessment of the toxicity of polyphenol-rich compounds with regard to cell type (Table 3). Naringin was found to be the least toxic polyphenol in all cell lines: its AUOC score was significantly lower than that of any other polyphenolic compound in at least one cell line. Aronox (in all the cell lines but HepG2) and the extracts from spent hop (in all the cell lines but Caco-2) and kale leaf (in A549, HMEC-1 and 3T3 cells) were also found to be very safe. Such an observation may be useful in undertaking the decision of using dietary supplements based on chokeberry, hop or kale leaf. Kaempferol and the extract from buckthorn bark were the most toxic polyphenols, and had the highest cumulative AUOC scores in all five cell lines. In addition, the effect of kaempferol on the analyzed cells was not significantly different from that observed for the extracts from walnut husk (toxic in all cell types but HepG2), mallow flower (mildly harmful in all cell lines) and silverweed herb (toxic in Caco-2, A549 and 3T3). Some polyphenol-rich compounds were found to be very harmful merely to selected cell lines: the extracts from oak leaf, hollyhock flower and oak bark were toxic to three of five cell lines, the extracts from rowan fruit and blackberry leaf were harmful to two of five cell lines, and the extracts from currant pomace and raspberry seed, Omnivir R, and resveratrol were detrimental to one of five tested cell lines. The cumulative normalized AUOC scores calculated across the assays, which ranged from –0.3 to –2.9 for the five least toxic and from 0.9 to 3.0 for the five most toxic polyphenol-rich compounds (Table 3), were further calculated across the lines (Table 7) to globally describe the cytotoxic properties of each examined plant compound; the averaged individual normalized AUOC scores were adjusted for either the effect of a cell line or for the effect of an assay or for both effects (Fig 5). However, two points need to be noted in this regard. First, attention should be paid to the analysis of correlation coefficients determined for each pair of methods within each cell line, the results of which suggest that the prioritization of chemicals based on global AUOC, herein employed to assess the toxicity ranking, is justified only to some extent: no statistically significant positive association was found between mitochondrial membrane potential or cell membrane permeability and nuclear area (Table 5). On the other hand, a significant positive correlation was detected between mitochondrial membrane potential and cell membrane integrity in all cell lines. Interestingly, a significant negative correlation was found between nuclear area and mitochondrial potential or cell membrane permeability, demonstrated in HMEC-1 cells, which clearly indicates that not every cytotoxic agent damaging mitochondrial and cell membrane compartments has to affect nuclear morphology and *vice versa*. Of note, the significant associations between assays or between cell lines were not compliant with the outcomes of tests comparing ‘sensitivities’ of assays or ‘sensitivities’ of cell lines, and such discrepancy most likely originates from the relatively high number of variables and small body of analyzed data. It is also worth emphasizing that both early and late cytotoxicity were evaluated in this study. Whereas the alterations of mitochondrial function occur early after cellular injury, changes in nuclear morphology and a loss of membrane integrity are recognized as biomarkers of acute or late-stage cell toxicity [5]. Therefore, our choice of assays, including those for the monitoring of both early and late cytotoxicity, undoubtedly proved to be beneficial; particularly so, when considering that different classes of natural extracts, chemicals or drugs may have a different sequence of effects on the cells [38]. Second, when verifying the possible impacts of an assay and a cell line on the resultant score values characterizing the global cytotoxicity, adjustments in cell line, an assay, or both were not found to have any considerable impact on the final outcome of the ranking sequence of toxicity. This observation indicates that neither the choice of a given cell line nor the choice of an assay mattered so much in ascending the prioritization of the sequence of polyphenol-rich compounds from the least to the most toxic.

### Conclusions

Overall, we propose an alternative approach for evaluating and hierarchically clustering various compounds according to their toxicity potential screened with the use of high content screening analysis. Such an algorithm should be recommended for a large number of compounds which induce a non-sigmoidal response, or agents with unknown toxicity/mechanism of action, particularly at an early stage of drug discovery. The suggested algorithm provides a rapid tool for identification of compounds with adverse effects, where the control agents are important factors in a successful and reliable analysis. Further testing of this approach with series of drugs with already known and differentiated toxicities is required to further strengthen the validation of the presented methodology.

## Supporting information

S1 Fig Representative dose-response curves for HMEC-1 cells treated with selected plant extracts/compounds in the study of mitochondrial membrane potential. (A) untransformed data for total area calculation (B) data after semi-log transformation for calculation of IC_50_.
(PDF)

S2 Fig Bland-Altman plots comparing various algorithms used for the evaluation of the extent of overall polyphenolic extract cytotoxicity. Plots representing the comparisons between: (A) global averaged normalized AOUC and individual normal scores averaged for extracts non-adjusted for assay and cell line, (B) global averaged normalized AOUC and averaged individual normal scores adjusted for both assay and cell line and (C) averaged individual normal scores non-adjusted for assay and cell line vs. averaged individual normal scores adjusted for both assay and cell line. For better legibility all variables were normalized to the same scale range of (0; 1). The limits for agreement between the used algorithms, marked by dashed red lines, estimated as the averaged difference between the compared indices ± 1.96* standard deviation of differences.
(PDF)

S3 Fig Mountain plots comparing various algorithms used for the evaluation of the extent of overall polyphenolic extract cytotoxicity. Individual normal scores averaged for tested polyphenolic extracts either non-adjusted or adjusted for assay and cell line were compared with the reference algorithm: global averaged normalized AOUC. (A) comparison of global averaged normalized AOUC *vs*. non-adjusted (blue line) or adjusted averaged individual normal scores (red line), (B) comparison of non-adjusted *vs*. adjusted averaged individual normal scores. The plot was created by evaluating the percentiles for ascending differences between the indices calculated for the compared algorithms. To generate the plot the numerical values of (100—percentile order) were assigned to the percentiles of the order above 50.
(PDF)

S1 Table AUOC calculation based on the raw data obtained after cell treatment with 0–50 μg/ml resveratrol. (PDF)

S2 TableRelationship between total area and IC_50_ values obtained from dose-response curves to resveratrol in MTT assay.Data were collected from human umbilical vein endothelial cells following 24-hour treatment with 1–200 μg/ml resveratrol, using 12 concentrations. Data are given for eight experiments, each performed with four repeats. Means and SD for total area estimated with a bootstrap-boosted calculus (1000 iterations). The IC_50_ values for individual repeats are given as mean ($\bar{x}$) and 95% confidence interval (95% CI). The last column contains the average IC_50_ values ($\bar{x}\;[-95\%CI;\;+95\%CI$) calculated for ‘pooled’ data of all four repeats.(PDF)
