# Supplementary material for: A new approach for the assessment of the toxicity of polyphenol-rich compounds with the use of high content screening analysis
Source: PLoS One. 2017 Jun 29;12(6):e0180022. doi: 10.1371/journal.pone.0180022 (PMC5491109; doi:10.1371/journal.pone.0180022)
Supplement: S1 Fig — (A) untransformed data for total area calculation (B) data after semi-log transformation for calculation of IC50. (PDF) [file pone.0180022.s001.pdf]

(A)

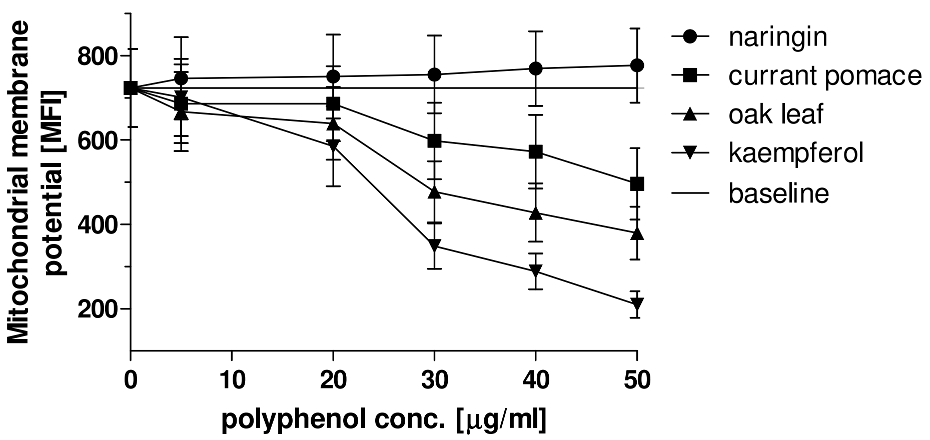

(B)

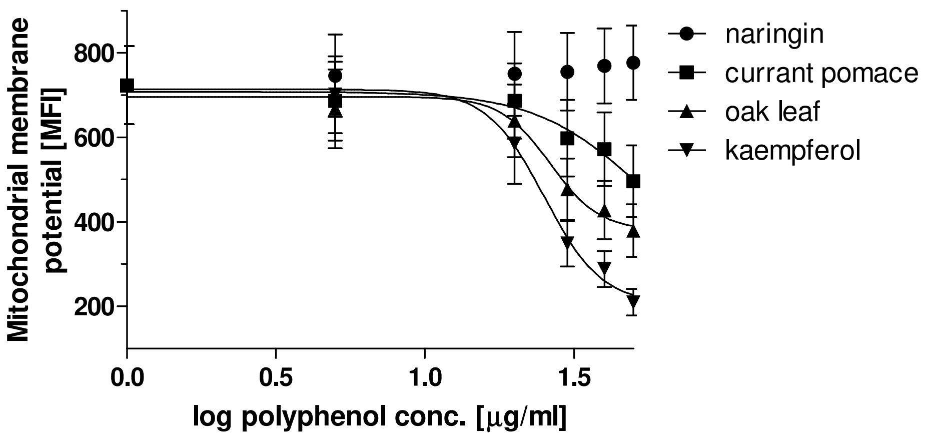

	naringin	currant pomace	oak leaf	kaempferol
**Total Area**	1619	4731	8745	12610
**IC_{50}**	Not converged	52.16	26.32	25.15

S1 Fig. Representative dose-response curves for HMEC-1 cells treated with selected polyphenol-rich compounds in the study of mitochondrial membrane potential. (A) untransformed data for total area calculation (B) data after semi-log transformation for calculation of IC_{50}.
