# Supplementary material for: A new approach for the assessment of the toxicity of polyphenol-rich compounds with the use of high content screening analysis
Source: PLoS One. 2017 Jun 29;12(6):e0180022. doi: 10.1371/journal.pone.0180022 (PMC5491109; doi:10.1371/journal.pone.0180022)
Supplement: S2 Fig — Plots representing the comparisons between: (A) global averaged normalized AOUC and individual normal scores averaged for extracts non-adjusted for assay and cell line, (B) global averaged normalized AOUC and averaged individual normal scores adjusted for both assay and cell line and (C) averaged individual normal scores non-adjusted for assay and cell line vs. averaged individual normal scores adjusted for both assay and cell line. For better legibility all variables were normalized to the same scale range of (0; 1). The limits for agreement between the used algorithms, marked by dashed red lines, estimated as the averaged difference between the compared indices ± 1.96* standard deviation of differences. (PDF) [file pone.0180022.s002.pdf]

A

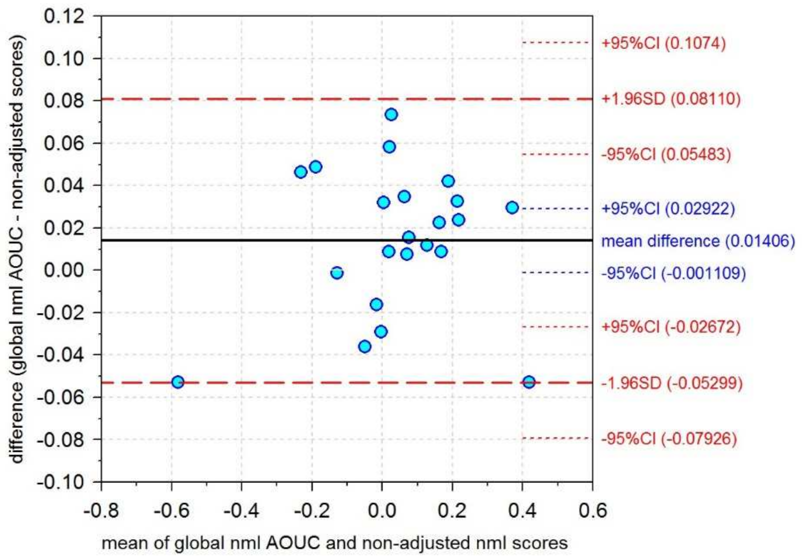
+95%CI (0.1074)
+1.96SD (0.08110)
-95%CI (0.05483)
+95%CI (0.02922)
mean difference (0.01406)
-95%CI (-0.001109)
+95%CI (-0.02672)
-1.96SD (-0.05299)
-95%CI (-0.07926)
difference (global nml AOUC - non-adjusted scores)
mean of global nml AOUC and non-adjusted nml scores

B

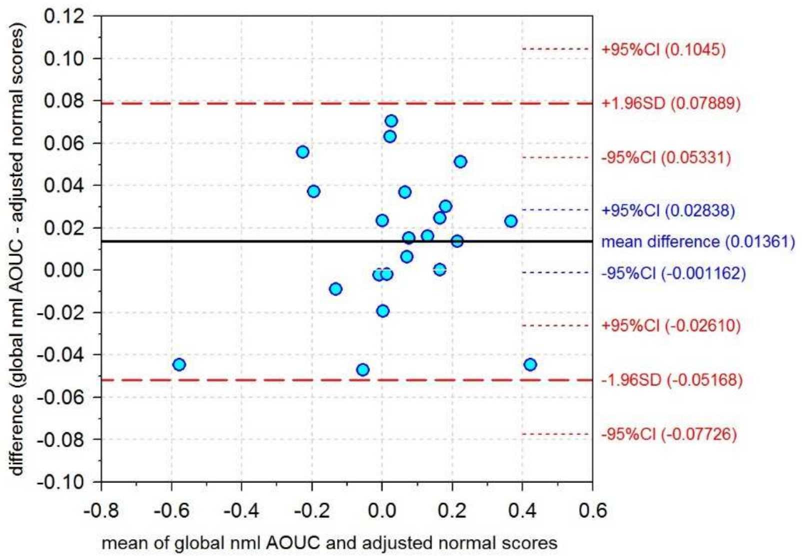
+95%CI (0.1045)
+1.96SD (0.07889)
-95%CI (0.05331)
+95%CI (0.02838)
mean difference (0.01361)
-95%CI (-0.001162)
+95%CI (-0.02610)
-1.96SD (-0.05168)
-95%CI (-0.07726)
difference (global nml AOUC - adjusted normal scores)
mean of global nml AOUC and adjusted normal scores

C

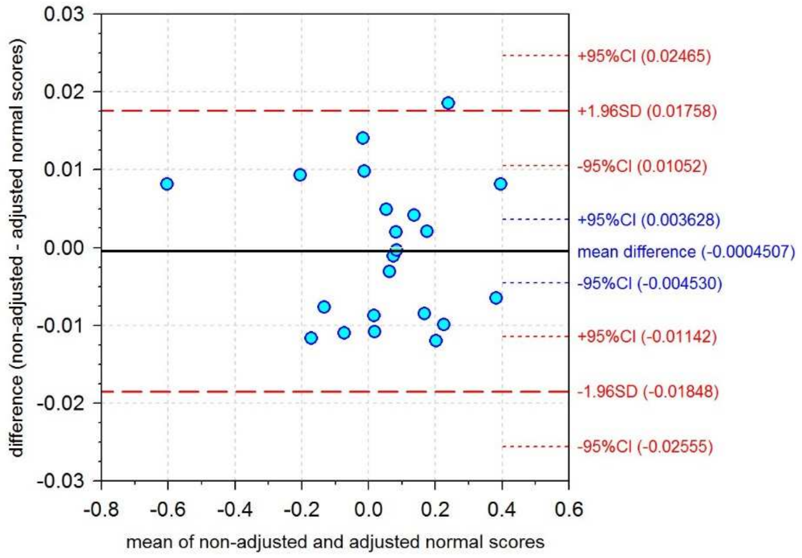
+95%CI (0.02465)
+1.96SD (0.01758)
-95%CI (0.01052)
+95%CI (0.003628)
mean difference (-0.0004507)
-95%CI (-0.004530)
+95%CI (-0.01142)
-1.96SD (-0.01848)
-95%CI (-0.02555)
difference (non-adjusted - adjusted normal scores)
mean of non-adjusted and adjusted normal scores

S2 Fig. Bland-Altman plots comparing various algorithms used for the evaluation of the extent of overall polyphenolic extract cytotoxicity. Plots representing the comparisons between: (A) global averaged normalized AOUC and individual normal scores averaged for extracts non-adjusted for assay and cell line, (B) global averaged normalized AOUC and averaged individual normal scores adjusted for both assay and cell line and (C) averaged individual normal scores non-adjusted for assay and cell line vs. averaged individual normal scores adjusted for both assay and cell line. For better legibility all variables were normalized to the same scale range of (0; 1). The limits for agreement between the used algorithms, marked by dashed red lines, estimated as the averaged difference between the compared indices ± 1.96* standard deviation of differences.
