# Supplementary material for: A new approach for the assessment of the toxicity of polyphenol-rich compounds with the use of high content screening analysis
Source: PLoS One. 2017 Jun 29;12(6):e0180022. doi: 10.1371/journal.pone.0180022 (PMC5491109; doi:10.1371/journal.pone.0180022)
Supplement: S3 Fig — Individual normal scores averaged for tested polyphenolic extracts either non-adjusted or adjusted for assay and cell line were compared with the reference algorithm: global averaged normalized AOUC. (A) comparison of global averaged normalized AOUC vs. non-adjusted (blue line) or adjusted averaged individual normal scores (red line), (B) comparison of non-adjusted vs. adjusted averaged individual normal scores. The plot was created by evaluating the percentiles for ascending differences between the indices calculated for the compared algorithms. To generate the plot the numerical values of (100—percentile order) were assigned to the percentiles of the order above 50. (PDF) [file pone.0180022.s003.pdf]

A

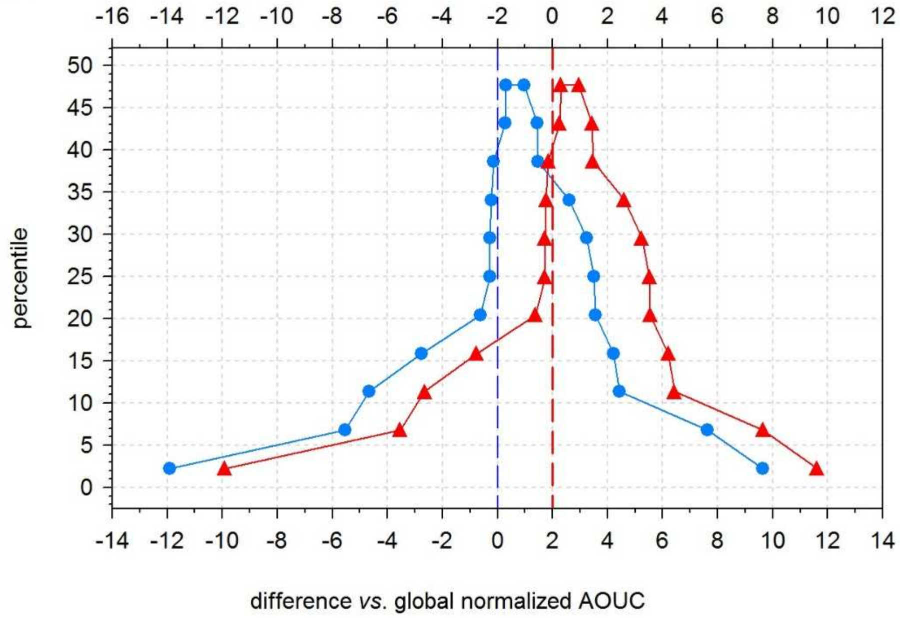

B

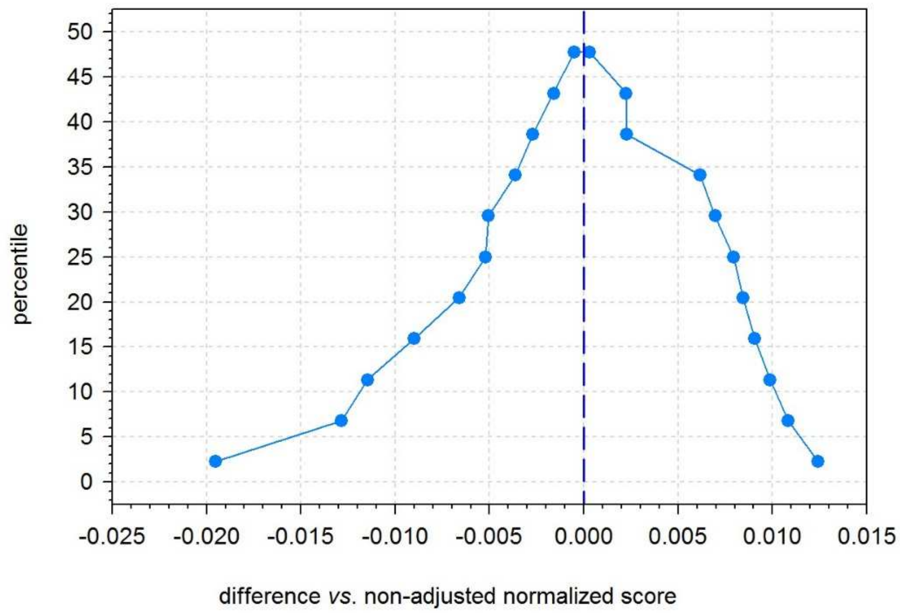

S3 Fig. Mountain plots comparing various algorithms used for the evaluation of the extent of overall polyphenolic extract cytotoxicity. Individual normal scores averaged for tested polyphenolic extracts either non-adjusted or adjusted for assay and cell line were compared with the reference algorithm: global averaged normalized AOUC. (A) comparison of global averaged normalized AOUC *vs*. non-adjusted (blue line) or adjusted averaged individual normal scores (red line), (B) comparison of non-adjusted *vs*. adjusted averaged individual

normal scores. The plot was created by evaluating the percentiles for ascending differences between the indices calculated for the compared algorithms. To generate the plot the numerical values of (100 - percentile order) were assigned to the percentiles of the order above 50.
