# Supplementary material for: A new approach for the assessment of the toxicity of polyphenol-rich compounds with the use of high content screening analysis
Source: PLoS One. 2017 Jun 29;12(6):e0180022. doi: 10.1371/journal.pone.0180022 (PMC5491109; doi:10.1371/journal.pone.0180022)
Supplement: S1 Table — (PDF) [file pone.0180022.s004.pdf]

S1 Table. AUOC calculation based on the raw data obtained after cell treatment with 0-50 µg/ml resveratrol.

Cell line	Number of experiement	mitochondrial potential			membrane permeability			nuclear area			Overall toxicity summed for all assays (C3+D3+E3)
		Total Area	Nml Score (vdW): Total Area	Mean Nml Score (vdW): Total Area	Total Area	Nml Score (vdW): Total Area	Mean Nml Score (vdW): Total Area	Total Area	Nml Score (vdW): Total Area	Mean Nml Score (vdW): Total Area	
**A**	**B**	**C1**	**C2**	**C3**	**D1**	**D2**	**D3**	**E1**	**E2**	**E3**	**F**
**HepG2**	1	1697	-1,05		446	-0,77		1116	1,30		
**HepG2**	2	1070	-1,66		1084	0,48		978	0,77		
**HepG2**	3	1834	-0,86		2618	1,05		856	0,22		
**HepG2**	4	2612	-0,34		404	-1,05		984	0,86		
**HepG2**	5	1699	-0,95	-0,97	564	-0,34	-0,13	916	0,48	0,72	**-0,38**
**Caco-2**	1	4422	-0,48		211	-0,62		3065	0,95		
**Caco-2**	2	4817	-0,28		142	-1,05		3423	1,05		
**Caco-2**	3	5185	-0,22		345	0,15		2979	0,86		
**Caco-2**	4	3369	-1,17		28	-1,97		3968	1,45		
**Caco-2**	5	5371	-0,09	-0,45	107	-1,30	-0,96	4306	1,97	1,26	**-0,14**
**A549**	1	6249	-0,77		168	-1,30		2935	1,30		
**A549**	2	6275	-0,69		188	-1,05		2630	1,17		
**A549**	3	7670	-0,41		195	-0,95		3374	1,97		
**A549**	4	7438	-0,48		150	-1,45		3283	1,66		
**A549**	5	8859	-0,15	-0,50	66	-1,97	-1,34	3157	1,45	1,51	**-0,34**
**HMEC-1**	1	6875	-0,09		59	-1,97		1343	1,17		
**HMEC-1**	2	8001	0,28		115	-1,45		1382	1,30		
**HMEC-1**	3	5396	-0,69		152	-0,86		1504	1,45		
**HMEC-1**	4	5736	-0,62		223	-0,48		1511	1,66		
**HMEC-1**	5	7146	0,09	-0,21	142	-0,95	-1,14	1809	1,97	1,51	**0,16**
**3T3**	1	4065	-0,34		1255	1,05		3563	0,69		
**3T3**	2	3597	-0,48		1710	1,45		5246	1,97		
**3T3**	3	3285	-0,62		1352	1,17		4210	0,86		
**3T3**	4	2927	-0,86		2117	1,97		5113	1,45		
**3T3**	5	4084	-0,28	-0,51	1575	1,30	1,39	5119	1,66	1,33	**2,20**
**Overall toxicity summed for all cell lines (sum of column C3 or D3 or E3)**				**-2,64**			**-2,18**			**6,32**	
**Global AUOC score (sum of column F)**											**1,50**
