# Supplementary material for: A new approach for the assessment of the toxicity of polyphenol-rich compounds with the use of high content screening analysis
Source: PLoS One. 2017 Jun 29;12(6):e0180022. doi: 10.1371/journal.pone.0180022 (PMC5491109; doi:10.1371/journal.pone.0180022)
Supplement: S2 Table — Data were collected from human umbilical vein endothelial cells following 24-hour treatment with 1–200 μg/ml resveratrol, using 12 concentrations. Data are given for eight experiments, each performed with four repeats. Means and SD for total area estimated with a bootstrap-boosted calculus (1000 iterations). The IC50 values for individual repeats are given as mean (x¯) and 95% confidence interval (95% CI). The last column contains the average IC50 values (x¯ [−95%CI; +95%CI) calculated for ‘pooled’ data of all four repeats. (PDF) [file pone.0180022.s005.pdf]

S2 Table. Relationship between total area and IC_{50} values obtained from dose-response curves to resveratrol in MTT assay. Data were collected from human umbilical vein endothelial cells following 24-hour treatment with 1–200 µg/ml resveratrol, using 12 concentrations. Data are given for eight experiments, each performed with four repeats. Means and SD for total area estimated with a bootstrap-boosted calculus (1000 iterations). The IC_{50} values for individual repeats are given as mean ($\bar{x}$) and 95% confidence interval (95% CI). The last column contains the average IC_{50} values ($\bar{x}\ [-95\%CI;\ +95\%CI)$ calculated for 'pooled' data of all four repeats.

number of experiment	repeat	Total Area	Total Area mean ± SD	IC_{50}	IC_{50} mean [95% CI]
1	1	32.0	33.6 ± 1.3	25.4 [17.1; 37.8]	32.7 [29.9; 35.7]
	2	33.6		33.9 [29.6; 38.8]	
	3	33.3		32.3 [25.0; 41.6]	
	4	35.5		35.4 [31.7; 39.5]	
2	1	40.2	34.8 ± 3.2	32.9 [22.1; 49.0]	30.7 [27.5; 34.2]
	2	34.1		32.8 [27.1; 39.7]	
	3	32.4		29.3 [25.2; 34.0]	
	4	32.5		30.6 [23.7; 39.4]	
3	1	56.3	60.9 ± 2.6	21.0 [11.8; 37.6]	21.9 [18.4; 26.1]
	2	62.5		19.2 [12.9; 28.8]	
	3	62.5		20.1 [13.5; 29.8]	
	4	62.3		27.0 [24.6; 29.8]	
4	1	64.1	63.0 ± 1.8	11.0 [6.5; 18.4]	15.3 [11.6; 20.2]
	2	65.0		20.4 [12.6; 32.8]	
	3	62.4		11.5 [5.4; 24.1]	
	4	60.4		21.2 [12.8; 35.0]	
5	1	51.5	50.6 ± 1.0	52.2 [49.6; 55.0]	52.3 [49.6; 55.1]
	2	51.2		~50.8 [very wide]	
	3	49.0		55.7 [43.4; 71.6]	
	4	50.8		~50.4 [very wide]	
6	1	38.5	30.9 ± 4.2	39.1 [33.0; 46.2]	40.4 [38.9; 42.0]
	2	29.2		41.0 [38.5; 43.6]	
	3	28.4		40.1 [37.6; 42.9]	
	4	28.2		40.7 [37.0; 44.9]	
7	1	48.1	44.6 ± 2.9	41.6 [35.5; 48.6]	39.8 [37.6; 42.1]
	2	43.2		40.6 [37.0; 44.5]	
	3	40.5		39.3 [34.1; 45.1]	
	4	46.8		38.5 [32.8; 45.1]	
8	1	35.7	30.1 ± 3.3	37.7 [33.9; 42.0]	39.6 [37.9; 41.4]
	2	28.8		40.9 [37.4; 44.8]	
	3	28.3		40.4 [37.0; 44.2]	
	4	27.5		39.5 [36.0; 43.4]	

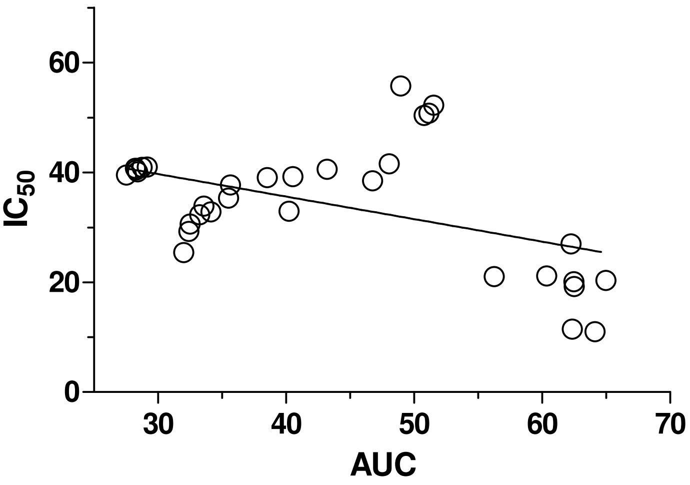

Number of XY Pairs	32
Spearman r	-0,409
95% confidence interval	-0,669 to -0,059
P value (one-tailed)	0,0101
P value summary	*
Exact or approximate P value?	Gaussian Approximation
Is the correlation significant? (alpha=0.05)	Yes
